# Astaxanthin-loaded brain-permeable liposomes for Parkinson’s disease treatment via antioxidant and anti-inflammatory responses

**DOI:** 10.1186/s12951-025-03104-8

**Published:** 2025-02-04

**Authors:** Thai-Duong Nguyen, Shristi Khanal, Eunhee Lee, Jinsol Choi, Ganesh Bohara, Nikesh Rimal, Dong-Young Choi, Soyeun Park

**Affiliations:** 1 College of Pharmacy, Keimyung University, 1095 Dalgubeoldae-Ro, Dalseo-Gu, Daegu, 42601 Republic of Korea; 2 College of Pharmacy, Yeungnam University, 280 Daehak-Ro, Gyeongsan, Gyeongbuk 38541 Republic of Korea

**Keywords:** Astaxanthin, Liposome, Lactoferrin, Parkinson’s disease, Dopamine

## Abstract

Parkinson’s disease is a neurodegenerative disorder characterized by cardinal motor symptoms resulting from the loss of dopaminergic neurons in the substantia nigra pars compacta. Although current medications may alleviate its symptoms, Parkinson’s disease remains incurable. Astaxanthin is an antioxidant and anti-inflammatory agent; however, its high susceptibility to oxidative degradation and low aqueous solubility limit its therapeutic efficacy. This study aimed to improve the pharmaceutical properties and neuroprotective effects of astaxanthin for Parkinson’s disease treatment by using lactoferrin-conjugated astaxanthin-loaded liposomes (Lf-ASX-LPs). We successfully formulated Lf-ASX-LPs with high encapsulation efficiency (97.6%) and favorable physical characteristics (diameter: 109.8 ± 1.1 nm; polydispersity index: 0.18 ± 0.01; zeta potential: − 9.5 ± 1.1 mV). Lf-functionalized liposomes demonstrated enhanced cellular uptake and permeation in a Transwell^®^ study, showing a 16.7-fold higher internalization in SH-SY5Y cells over 24 h than those without Lf conjugation. Additionally, Lf functionalization enhanced brain penetration ability, as demonstrated by a biodistribution study using nude mice, compared to LPs without Lf conjugation. In vitro, Lf-ASX-LPs reduced cell loss by 20.1% and oxygen species by 30.0%, ameliorated the reduction in mitochondrial membrane potential under 1-methyl-4-phenylpyridinium-induced toxicity by 40.1%, and reduced extracellular nitric oxide levels under lipopolysaccharide-induced toxicity by 32.0%, indicating cytoprotective and antioxidant effects. In a 1-methyl-4-phenyl-1,2,3,6-tetrahydropyridine (MPTP)-induced Parkinson’s disease mouse model, treatment with Lf-ASX-LPs resulted in 5.0-, 3.1-, and 5.6-fold increases in dopamine levels, TH^+^ fiber density, and TH^+^ neurons, respectively, restoring dopaminergic neuron damage. The developed formulation also alleviated behavioral impairment and neuroinflammation, reducing astrocyte and microglial activation in the striatum and substantia nigra of the MPTP-treated animals. Thus, our formulation of Lf-ASX-LPs represents a promising strategy for providing neuroprotection and retarding Parkinson’s disease progression.

**Graphical abstract:**

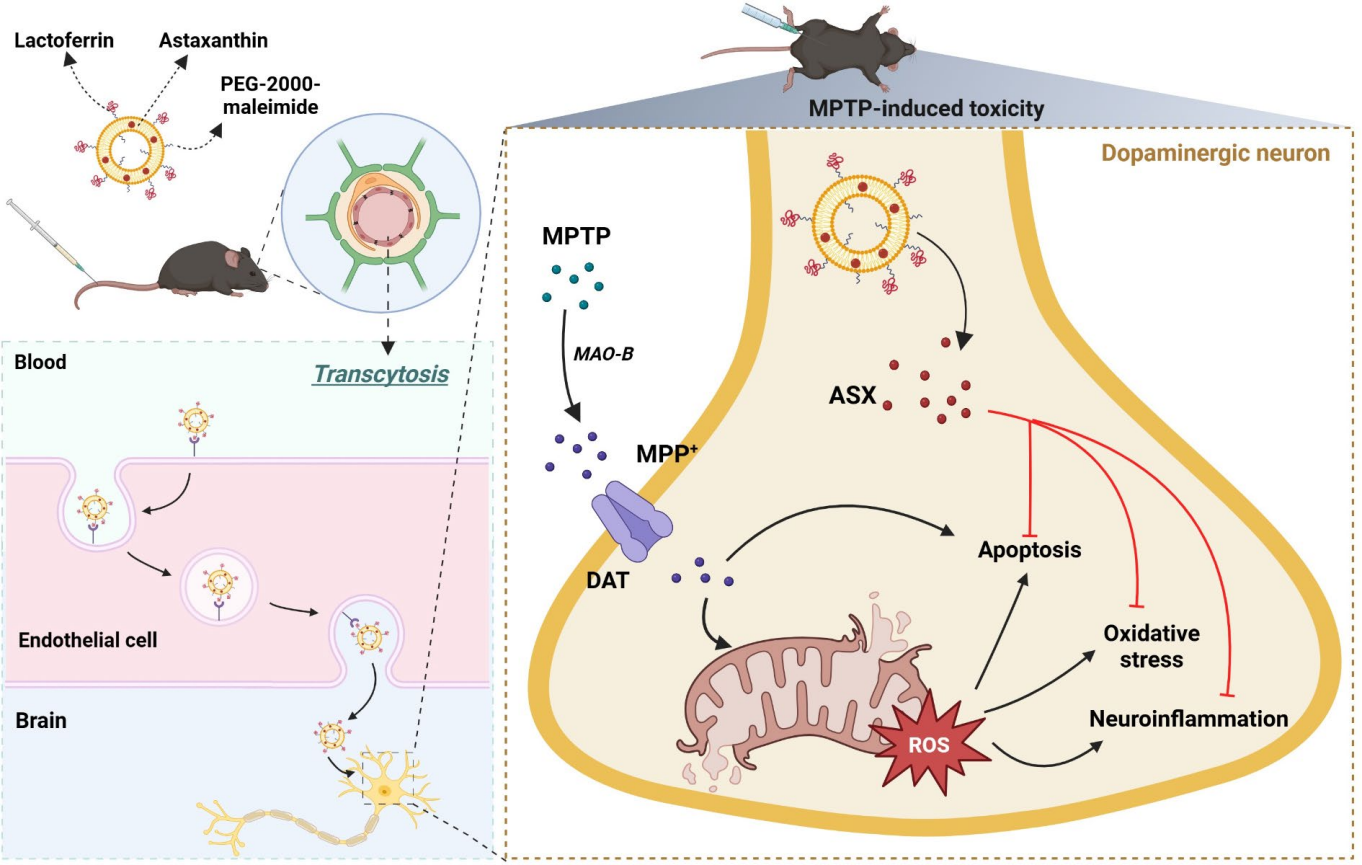

**Supplementary Information:**

The online version contains supplementary material available at 10.1186/s12951-025-03104-8.

## Introduction

Parkinson’s disease (PD) is the second most common neurodegenerative disorder after Alzheimer’s disease. Currently, more than 10 million people worldwide suffer from PD [1], with a continuously increasing PD population placing a huge burden on global public health care systems. The motor symptoms of PD include bradykinesia, rigidity, and resting tremor associated with disruption of dopaminergic neurons in the substantia nigra. The impaired dopamine (DA) release triggered by the loss of dopaminergic neurons affects muscle contraction and movement coordination [2, 3]. Notably, the symptoms deteriorate over time, markedly reducing the quality of later life. Although current pharmacotherapies mitigate the symptoms of PD, they neither halt disease progression nor cure it [4]. Thus, more effective therapeutics are urgently needed for PD treatment.

Degeneration of the dopaminergic neural network in the substantia nigra can be triggered by various factors, such as α-synuclein aggregation, oxidative stress, mitochondrial dysfunction, and neuroinflammation. Recently developed anti-inflammation agents have shown promising results against PD. In PD pathogenesis, neuroinflammation characterized by microglial activation, cytokine production, and oxidative stress plays a pivotal role in the progressive degeneration of dopaminergic neurons [5, 6]. Astaxanthin (ASX, 3,3′-dihydroxy-β-carotene-4,4′-dione) has recently emerged as a more potent antioxidant than other common antioxidants such as β-carotene, vitamin C, and vitamin E [7]. ASX also modulated inflammatory responses in neurodegenerative diseases by downregulating proinflammatory cytokines via attenuation of NF-κB activity [8, 9]. Wang et al. reported that ASX mitigated oxidative stress, apoptotic cell death, behavioral impairment, and dopaminergic neuron loss in a paraquat-induced PD mouse model [10]. The compound showed a good safety profile at an oral dose of 1,240 mg/kg/day for 90 days with no adverse reactions in rats [11]. A randomized clinical trial demonstrated that daily administration of 6 mg of ASX-enriched extract for 8 weeks or 20 mg for 4 weeks had no significant effects on the blood pressure or chemistry parameters of healthy volunteers [12]. However, a high daily dose of ASX (30 mg/kg) should be applied over an extended period to achieve neuroprotective effects because of the limited bioavailability of free ASX, which is closely related to its low solubility in water [13, 14]. Furthermore, ASX is unstable due to its conjugated double bond and can easily be degraded by several environmental factors, including light, heat, oxygen, and pH.

To address the limitations imposed by its poor solubility, nanocarriers such as liposomes (LPs) are needed to deliver hydrophobic ASX into the brain. LPs are nanoscale structures consisting of a phospholipid bilayer and aqueous core that can encapsulate both hydrophobic and hydrophilic compounds [15]. LPs made of synthetic phospholipids are biocompatible and biologically inert, with low systemic toxicity. In recent years, LPs have been used as carriers of therapeutic or imaging agents for the treatment or diagnosis of disorders in the central nervous system (CNS) [16–18]. Compared with conventional LPs, cationic LPs can be transported across the blood–brain barrier (BBB) via electrostatic interaction with the negative charge of the luminal surface of the BBB, known as adsorptive-mediated transcytosis [19]. To enhance delivery efficacy, cationic LPs are commonly administered intra-arterially, though this may cause various issues and rapid clearance. PEGylated LPs are an alternative strategy due to the protection afforded by non-specific plasma protein absorption and its uptake in the reticuloendothelial system, resulting in extended systemic circulation and improved biodistribution in the brain. However, both cationic and PEGylated LPs have no specific binding affinity for crossing the BBB. Therefore, LPs should be modified with ligands for appropriate BBB receptors to enhance their penetration into the brain [20, 21].

Receptor-mediated transcytosis is the primary physiological pathway for transporting biological macromolecules across the BBB. Antibodies or peptides that bind to specific receptor-mediated transcytosis receptors may be effective ligands for use in nanocarriers targeting the CNS [22]. Numerous studies have shown that lactoferrin (Lf) receptors (LfRs), expressed on the capillary endothelial cells of the brain, transport Lf across the BBB through transcytosis [23, 24]. Lf, a transferrin (Tf) family member, consists of a single iron-binding glycoprotein chain with 690 amino acids. Liu et al. demonstrated that more Lf-modified DiR-loaded LPs reached the brain than bare DiR-loaded LPs in a glioma-bearing ICR mice model [25]. Rodrigues et al. recently designed dual-modified LPs containing Tf ligands that improved the efficient delivery of plasmid DNA into the mouse brain [26]. However, the uptake efficiency of Lf is markedly higher than that of Tf due to the strong affinity of positively charged Lf for LfRs [27]. More importantly, LfR is overexpressed in neurodegenerative diseases such as Alzheimer’s, PD, and Huntington’s disease [28]. Thus, Lf is a promising candidate ligand for brain-targeted drug delivery.

In this study, we assessed the performance of ASX-loaded LPs (ASX-LPs) formulated using the thin-film hydration method and conjugated with Lf through a thiol-maleimide coupling reaction in the treatment of PD. We hypothesized that Lf-conjugated LPs would show enhanced permeation through the BBB. We investigated the antioxidant and anti-inflammatory activities of Lf-conjugated ASX-LPs (Lf-ASX-LPs) and their mitigating effects on dopaminergic neurodegeneration in a 1-methyl-4-phenyl-1,2,3,6-tetrahydropyridine (MPTP)-mediated PD mouse model. To the best of our knowledge, this is the first study to demonstrate the efficacy of Lf-ASX-LPs in attenuating Parkinsonian neurodegeneration.

## Materials and methods

### Materials

ASX, recombinant human Lf, 1,2-dioleoyl-*sn*-glycero-3-phosphocholine (DOPC), cholesterol, lipopolysaccharide (LPS), modified Griess reagent, and selegiline hydrochloride were bought from Sigma-Aldrich (St. Louis, MO, USA); 1,2-dipalmitoyl-*sn*-glycero-3-phosphocholine (DPPC), 1,2-distearoyl-*sn*-glycero-3-phosphoethanolamine-N-[maleimide(polyethylene glycol)-2000] (DSPE-PEG-maleimide), 2′,7′-dichlorofluorescein diacetate (DCFH-DA), and 1,2-dioleoyl-*sn*-glycero-3-phosphoethanolamine-N-(cyanine 5.5) (DOPE-Cy5.5) were purchased from Avanti Polar Lipids (Alabaster, AL, USA); 1-methyl-4-phenylpyridinium (MPP^+^) was obtained from Cayman Chemicals (Ann Arbor, MI, USA); MPTP was obtained from TCI (Tokyo, Japan); Oregon Green™ 488 1,2-dihexadecanoyl-sn-glycero-3-phosphoethanolamine (OG488-DHPE), a Pierce bicinchoninic acid (BCA) protein assay kit, 2-iminothiolane hydrochloride (Traut’s reagent), 5,5-dithio-bis-2-nitrobenzoic acid (Ellman’s reagent), antibodies to glial fibrillary acidic protein (GFAP), and tyrosine hydroxylase (TH) were purchased from Thermo Fisher Scientific (Waltham, MA, USA); antibody to ionized calcium-binding adaptor molecule 1 (Iba-1) was bought from Wako Pure Chemical Corporation (Osaka, Japan); 5,5,6,6′-tetrachloro‐1,1′,3,3′‐tetraethylbenzimi‐dazoylcarbocyanine iodide (JC-1) dye, 3-(4,5-dimethylthiazol-2-yl)-2,5-diphenyltetrazolium bromide (MTT), and human lactoferrin ELISA kits were obtained from Abcam (Cambridge, UK); biotinylated secondary antibody, avidin–biotin–peroxidase complex, and Vectashield mounting medium were obtained from Vector Laboratories (Burlingame, CA, USA); and antibodies against IL-1β, TNF-α, and β-actin were bought from Cell Signaling Technology (Tokyo, Japan), GeneTex (Irvine, CA, USA), and AbFrontier (Seoul, Republic of Korea), respectively.

## Synthesis of liposomes

**Fig. 1:**
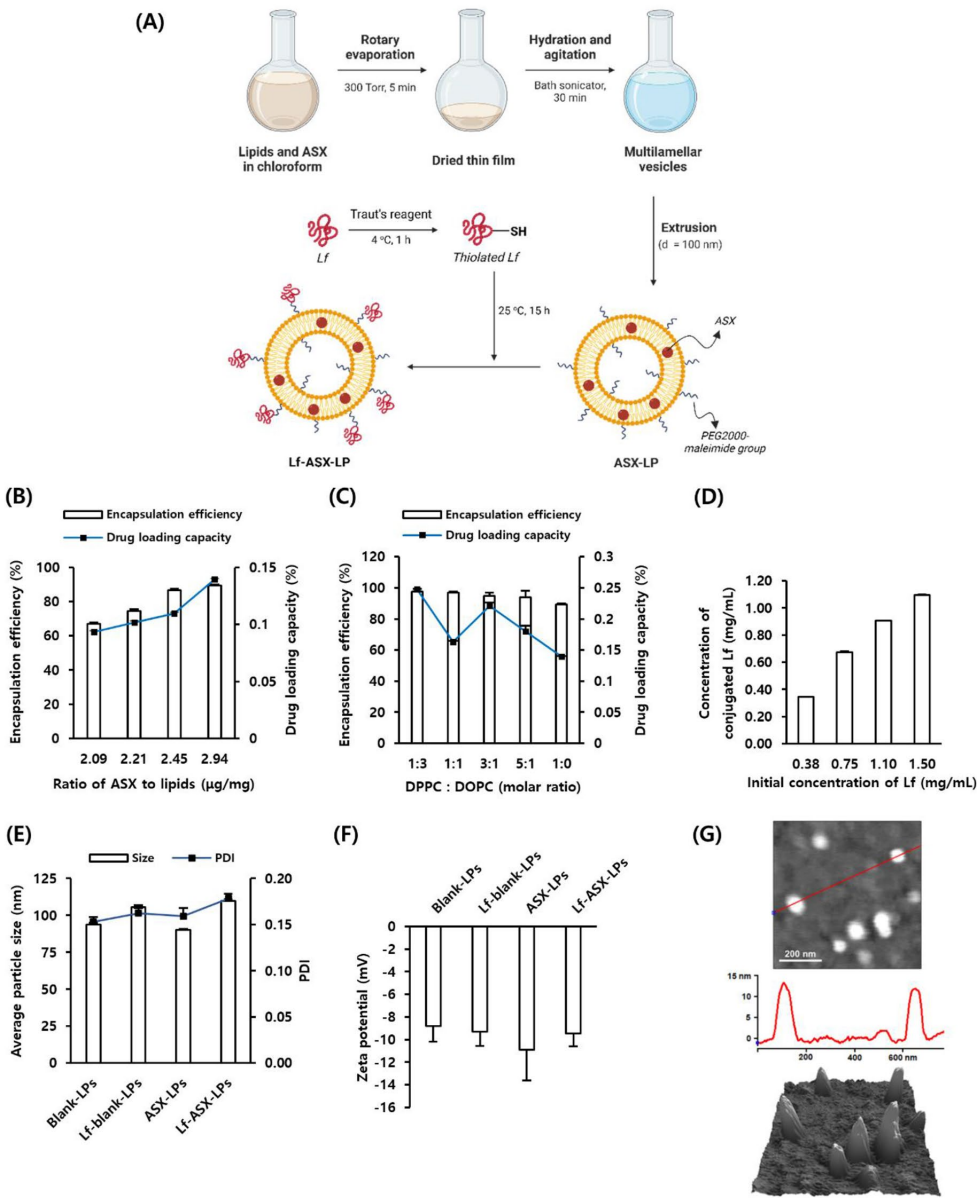
Optimization of ASX encapsulation and characterization of LPs. (**A**) Schematic diagram of Lf-ASX-LP preparation. EE and LC of ASX-LPs were measured under varying (**B**) ASX-to-lipid ratios and (**C**) lipid compositions. (**D**) Concentration of conjugated Lf on LPs under varying initial concentrations of Lf. (**E**) Average particle size, PDI, and (**F**) zeta potential of each formulation. (**G**) Topographic images of Lf-ASX-LPs obtained by AFM

LPs were created using the thin-film hydration method (Fig. 1A). Briefly, LPs were prepared using DPPC, DOPC, cholesterol, and DSPE-PEG-maleimide at a molar ratio of 20:60:30:0.5. 0.5 mol% of either OG488-DSPE or DOPE-Cy5.5 was added for the in vitro Transwell^®^ and in vivo biodistribution tests, respectively. The lipids and ASX were dissolved in chloroform in a round-bottom flask. The chloroform was evaporated using a rotary evaporator under a vacuum at 40 °C to form a thin film (N-1200B, EYELA, Bohemia, NY, USA). The lipid film was hydrated using phosphate-buffered saline (PBS, pH 7.4), followed by sonication for 40 min. To obtain LPs, multilamellar vesicles were extruded using a polycarbonate membrane with a pore size of 100 nm (Avanti Polar Lipids).

Lf was conjugated into LPs using Traut’s reagent (in 0.1 M HEPES buffer at pH 8.0 containing 2 mM EDTA and 1 mM MgCl_2_) for coupling in an ice bath over 1 h with gentle stirring. Lf was then purified through PD-10 desalting columns (Cytiva, Marlborough, MA, USA) equilibrated with PBS. The thiol concentration was measured with Ellman’s reagent. Maleimide-functionalized LPs were immediately mixed with purified Lf and incubated overnight at 25 °C. Unreacted ASX and Lf were removed by dialysis overnight at 4 °C using a 300-kDa molecular weight cutoff (MWCO) dialysis membrane (Spectrum Labs, Rancho Dominguez, CA, USA). The final LPs in PBS at pH 7.4 were stored at 4 °C for further experiments.

## Characterization of liposomes

The average particle size and polydispersity index (PDI) were determined using dynamic light scattering, while zeta potential was measured using laser Doppler electrophoresis (Otsuka ELSZ-2000 Series, Otsuka Electronics, Osaka, Japan). To ensure the physical stability of our formulations, we have conducted the time-elapsed measurements of DLS at the storage (4 °C) and physiological (37 °C) temperature. The morphology of Lf-ASX-LPs was examined using atomic force microscopy with tapping mode imaging (MFP3D^®^, Asylum Research, Santa Barbara, CA, USA).

To quantify the encapsulation efficiency (EE, Eq. 1) and loading capacity (LC, Eq. 2) of ASX, free ASX was removed using the dialysis method. The particles were dissolved in a mixture of dimethyl sulfoxide and ethanol and then sonicated for 5 min to break down the liposomal structure and release encapsulated ASX, followed by centrifugation for 5 min at 13 500 *×g*. Mean ASX concentration was measured at an absorbance of 480 nm using UV–Vis spectroscopy (Shimadzu UV-1800, Kyoto, Japan) from three measurements.

1 $${\rm{EE}}\left( \% \right){\rm{ = }}{\matrix{{\rm{Amount}}\>{\rm{of}}\>{\rm{encapsulated}}\> \hfill \cr {\rm{ASX}}\>{\rm{after}}\>{\rm{dialysis}} \hfill \cr} \over \matrix{{\rm{Total}}\>{\rm{amount}}\>{\rm{of}}\> \hfill \cr {\rm{ASX}}\>{\rm{before}}\>{\rm{dialysis}} \hfill \cr} } \times {\rm{100}}\%$$

2 $${\rm{LC}}\>\left( \% \right)\>{\rm{ = }}{\matrix{{\rm{Amount}}\>{\rm{of}}\>{\rm{encapsulated}} \hfill \cr {\rm{ASX}}\>{\rm{after}}\>{\rm{dialysis}} \hfill \cr} \over {{\rm{Total}}\>{\rm{amount}}\>{\rm{of}}\>{\rm{lipids}}\>{\rm{in}}\>{\rm{LPs}}}} \times {\rm{100}}\%$$

A BCA protein assay was conducted to measure the conjugation efficiency (CE, Eq. 3) of Lf. Briefly, free Lf was removed using centrifugation with an ultracentrifugal filter (100 kDa MWCO, Amicon^®^) for 1 h at 4 500 *×g*. Then, 50 µL of BCA working reagent was added to 1 mL of diluted Lf-conjugated LPs and incubated for 30 min at 37 °C. Absorbance was measured at 562 nm using UV–Vis spectroscopy. The concentration of conjugated Lf was calculated using a standard calibration curve.

3 $${\rm{CE}}\left( \% \right)\>{\rm{ = }}{{{\rm{Amount}}\>{\rm{of}}\>{\rm{conjugated}}\>{\rm{Lf}}} \over {{\rm{Total}}\>{\rm{amount}}\>{\rm{of}}\>{\rm{Lf}}}} \times {\rm{100}}\%$$

The bioactivity of Lf in the final Lf-ASX-LPs was measured using a human lactoferrin ELISA Kit (Abcam). LPs stored at 4 °C were tested for stability at 1, 2, 3, 4, 7, 14, 21, and 28 d. All evaluations were conducted in triplicate.

The cumulative release study of ASX was conducted using the dialysis membrane (MWCO 10 kDa) method. The release medium (PBS at pH 7.4) was maintained at 37 °C and supplemented with 0.5% (v/v) Tween 80 to maintain sink conditions. At certain time intervals, the sampled solution was replaced with the same volume of fresh medium. The concentration of ASX released into the medium was quantified by UV–Vis spectroscopy at 480 nm. Accelerated stability testing was also conducted to ensure the chemical stability of ASX encapsulated in LPs. Briefly, Lf-ASX-LPs and ASX suspension in 5% dimethyl sulfoxide and 5% Tween 80 were stored at 45 °C and 60 °C. The concentration of ASX from these formulations was determined at various time points.

### In vitro evaluation of liposomes

#### Cell viability test

Cell viability was measured using MTT assay. Briefly, human brain microvascular endothelial cells (HBMECs), SH-SY5Y, and BV-2 cells were grown in 96-well plates at a density of 4 $\:\times\:$ 10^4^ cells/well for 24 h. The cells were treated with blank LPs, free ASX, ASX-LPs, and Lf-ASX-LPs (equivalent to 18 µM of ASX) for 6 h, washed with PBS, and incubated in medium containing 0.5 mg/mL MTT for 4 h. The medium was aspirated gently and replaced with 100 µL DMSO per well to dissolve the formazan crystals. Absorbance at 570 nm was measured using a microplate reader (Infinite M200 Pro, Tecan Austria GmbH, Grodig, Austria) in triplicate. The cell viability (%) was calculated as (*A*_1_/*A*_0_) × 100%, where *A*_0_ is the absorbance of the control and *A*_1_ is the absorbance of the samples.

#### In vitro BBB penetration and cellular uptake of Lf-conjugated LPs

For the BBB penetration study, Transwell^®^ inserts (0.4 μm pore size; Corning, Corning, NY, USA) were coated with rat tail collagen type I (Discovery Labware, Bedford, MA, USA). The HBMECs were cultivated at a density of 5 × 10^4^ cells/well on the upper insert for 3 d, and the media were replaced with those containing OG488-LPs and Lf-OG488-LPs. The medium in the basolateral chamber was collected at different time points and fluorescence signals were recorded using a microplate reader (FLUOstar Omega, BMG Labtech, Ortenberg, Germany) at excitation/emission wavelengths of 480 nm/530 nm, respectively.

**Fig. 2:**
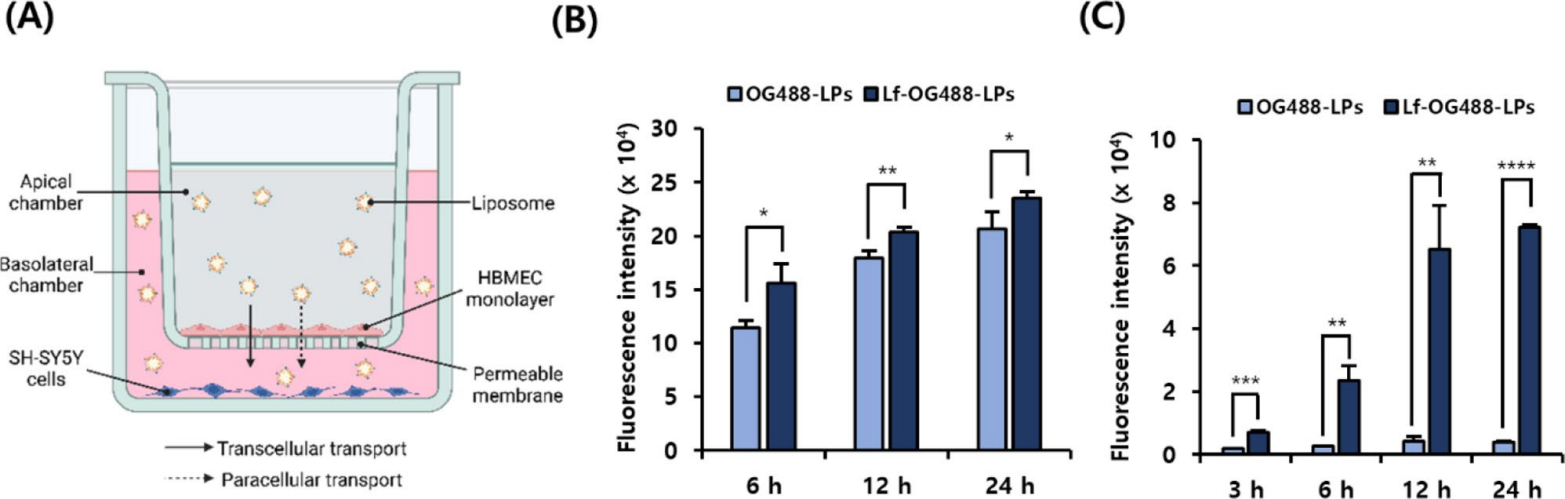
In vitro permeation experiments. (**A**) Schematic representation of Transwell^®^ study with HBMECs and SH-SY5Y cells. (**B**) Mean fluorescence intensity of medium in the basolateral chamber at 6, 12, and 24 h of incubation with OG488-LPs or Lf-OG488-LPs. (**C**) Cellular uptake of OG488-LPs and Lf-OG488-LPs by SH-SY5Y cells in the basolateral chamber at 3, 6, 12, and 24 h. All data are presented as the mean ± SD (*n* = 3). *p < 0.05, **p < 0.01, ***p < 0.001, ****p < 0.0001

A co-culture model of HBMECs and SH-SY5Y cells was established to determine the BBB penetration and cellular uptake of the Lf-conjugated LPs labeled with OG488. Briefly, HBMECs were seeded as mentioned above, while SH-SY5Y cells were seeded at 1 × 10^5^ cells/well on the lower compartments of the Transwell^®^ insert for 24 h (Fig. 2A). Then media were replaced with those containing OG488-LPs or Lf-OG488-LPs. SH-SY5Y cells were harvested at 3, 6, 12, and 24 h to evaluate the time-dependent cellular uptake of Lf-conjugated LPs using flow cytometry (FACS, BD Biosciences, La Jolla, CA, USA).

#### Cytoprotective activity against MPP^+^-induced cytotoxicity

SH-SY5Y cells grown in 96-well plates at a density of 4 × 10^4^ cells/well for 24 h were pretreated with blank LPs, free ASX, ASX-LPs, or Lf-ASX-LPs (equivalent to 18 µM ASX) for 6 h, incubated with 2 mM MPP^+^ for 24 h, and cell viability was measured using the MTT assay.

#### Evaluation of intracellular antioxidant activity

DCFH-DA dye was used to measure the increase in intracellular reactive oxygen species (ROS) levels under exposure to MPP^+^. SH-SY5Y cells were cultivated on black 96-well plates coated with type I collagen at a density of 4 × 10^4^ cells/well for 24 h. The cells were then pretreated with the test agents (equivalent to 18 µM ASX) for 6 h before being exposed to 1 mM MPP^+^ solution for 24 h. After removing the existing medium, the cells were incubated with a medium containing 20 µM DCFH-DA dye for 30 min in the dark. The cells were washed with PBS and the fluorescence intensity of intracellular dichlorofluorescein (DCF) was measured using the FLUOstar Omega microplate reader.

#### Determination of mitochondrial membrane potential (MMP)

JC-1 dye was used to measure the reduction in MMP levels after exposure to MPP^+^. SH-SY5Y cells were cultured and treated in the same way as for the antioxidant test, exposed to 2 mM MPP^+^ for 24 h, and treated with fresh media containing 20 µM of JC-1 dye for 15 min in the dark. The cells were rinsed with PBS twice to remove excessive JC-1 dye and the FLUOstar Omega microplate reader was used to measure the fluorescence intensity of JC-1 monomers (488 nm/530 nm of excitation/emission) and aggregates (530 nm/590 nm of excitation/emission). The results are presented as the ratio of JC-1 aggregates: monomers.

#### Quantification of extracellular nitric oxide levels

The extracellular nitric oxide (NO) levels were indirectly measured using a Griess assay to determine the production of nitrite in the culture medium. BV-2 cells were first seeded at a density of 1 × 10^5^ cells/well in a 24-well plate for 24 h, pretreated with the test agents (equivalent to 18 µM ASX) for 6 h, and incubated with medium containing 500 ng/mL LPS, except for the control group. After 24 h incubation, the culture medium was collected and centrifuged for 10 min at 12 000 rpm. We mixed 100 µL of the supernatant with 100 µL of modified Griess reagent in a 96-well plate. The reactions were kept at 25 °C for 15 min and absorbance at 540 nm was measured (Infinite M200 Pro). Freshly prepared NaNO_2_ solution was used as a standard for calculating nitrite levels.

### In vivo evaluation of Lf-ASX-LPs

#### Animals

We allowed 7-week-old BALB/c nude and 8-week-old C57BL/6 mice (male; 20–25 g; Hyochang Science, Seoul, Republic of Korea) to be acclimatized over 1 week. The mice were housed in micro-isolator cages under a 12-h light/dark cycle with free access to food and water, in compliance with protocols reviewed and approved by the Institutional Animal Care and Use Committee of Keimyung University (KM2023-017).

#### Biodistribution of Lf-cyanine 5.5 (Cy5.5)-LPs

BALB/c nude mice were assigned to four groups (10 mice/group) and intravenously injected with saline, free Cy5.5, Cy5.5-loaded LPs (Cy5.5-LPs), or Lf-Cy5.5-LPs. Under isoflurane-induced anesthesia, each mouse was imaged in vivo using VISQUE™ InVivo Elite (Vieworks, Anyang, Republic of Korea) at 1, 3, 6, and 24 h post-injection. For the ex vivo experiment, the animals were then sacrificed using CO_2_ asphyxiation at 4–24 h post-injection. The fluorescence intensity was measured for the major organs, including the brain, heart, lungs, liver, spleen, and kidneys.

#### Dose-escalation study

**Scheme 1:**
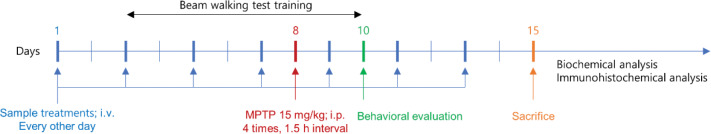
Experimental workflow for animal study

C57BL/6 mice were divided into five groups (5 mice/group) receiving various doses of Lf-ASX-LPs via intravenous injection: Group 1 (control) received saline; Group 2 received dose 1 of Lf-ASX-LPs (equivalent to 0.133 mg ASX/kg) and MPTP; Group 3 received dose 2 of Lf-ASX-LPs (equivalent to 0.267 mg ASX/kg) and MPTP; Group 4 received dose 3 of Lf-ASX-LPs (equivalent to 0.400 mg ASX/kg) and MPTP; and Group 5 received dose 4 of Lf-ASX-LPs (equivalent to 0.665 mg ASX/kg) and MPTP. Each group received injections every other day for a total of seven times (Scheme 1). On day 8, groups 2, 3, 4, and 5 were injected intraperitoneally with 15 mg/kg MPTP four times at 1.5-h intervals. Behavioral evaluations were conducted on day 10. The mice were sacrificed using CO_2_ asphyxiation on day 15. For immunohistochemistry (IHC) staining, the right hemisphere of each brain was collected and fixed in 4% paraformaldehyde. After 2 d of storage at 4 °C, the samples were rinsed and immersed in 30% sucrose solution. For neurochemical analysis, striatum tissues were collected from the left hemisphere and kept in a deep freezer. The levels of DA and its metabolite were measured the next day.

#### Neuroprotection in MPTP-induced model

C57BL/6 mice were divided into nine groups (9 mice/group) receiving different treatments: Group 1 received normal saline; Group 2 received MPTP; Group 3 received selegiline (SEL, 10 mg/kg/dose) and MPTP; Group 4 received free ASX; Group 5 received free ASX and MPTP; Group 6 received Lf-blank-LPs; Group 7 received Lf-blank-LPs and MPTP; Group 8 received Lf-ASX-LPs; and Group 9 received Lf-ASX-LPs and MPTP. ASX and Lf-ASX-LPs were administered at doses equivalent to 0.665 mg ASX/kg/dose. The experimental workflow was similar to that for the dose-escalation experiment (Scheme 1).

#### Behavioral test

The beam walking, challenging walking, and cylinder tests were conducted 2 d after MPTP injection, as previously described [29]. Each test comprised three trials per mouse and was recorded using a video camera. Briefly, for the beam walking test, a 100-cm wooden beam consisting of four equal-length sections with gradually decreasing widths (from 3.5 cm to 0.5 cm) was placed across two overturned cages. The narrower end of the beam was placed in the home cage (Supplementary Fig. S1A). The duration required to reach the home cage was recorded. Before the actual assessment on day 10, all mice were pre-trained for 1 week to traverse the beam toward the home cage.

A 1-cm^2^-mesh grid with a width corresponding to the beam width was placed 1 cm above the beam surface for the challenging walking test (Supplementary Fig. S1B). We measured the duration required for the mouse to reach the home cage while traversing and passing through the grid. The number of foot slips per step was calculated from recorded videos.

The cylinder test was performed to investigate the exploratory behavior of mice using a transparent glass cylinder (12 cm diameter and 20 cm height). The number of rearings, when mice raise their forelimbs and touch the cylinder walls to support their weight, was recorded for 3 min using the video camera. We counted the number of touches with one or both forelimbs [30, 31].

#### Quantification of DA and 4-dihydroxy phenylacetic acid (DOPAC)

We analyzed the levels of DA and DOPAC in the striatum using a high-performance liquid chromatography instrument (HPLC) (1260 Infinity, Agilent Technologies, Santa Clara, CA, USA) with an electrochemical detector (Coulochem III, Thermo Fisher Scientific) [32]. Briefly, the pre-weighed striatal tissues were homogenized in a 0.1 N perchloric acid solution, centrifuged at 13 000 *×g* and 4 °C for 30 min, and eluted using a mobile phase consisting of 17% acetonitrile, 100 µL/L triethylamine, 75 mM NaH_2_PO_4_, 1.7 mM 1-octane sulfonic acid, and 25 µM EDTA. Each sample was injected and isocratically eluted at a flow rate of 0.6 mL/min.

#### Immunohistochemical staining

Coronal brain sections (thickness of 30 μm) were prepared using a sliding microtome (Microm HM 450, Thermo Fisher Scientific, Walldorf, Germany). The sections were rinsed of the cytoprotectant solution and incubated with 3% H_2_O_2_ for 20 min, washed five times with KPBS, and stained with rabbit polyclonal primary antibody against TH (1:3,000), GFAP (1:3,000), or Iba-1 (1:1,000) in KPBS containing 0.4% Triton X-100 at 4 °C overnight. The next day, the sections were treated with biotinylated secondary antibody (1:1,000) and incubated in avidin–biotin–peroxidase complex for 1 h. We used 3,3-diaminobenzidine solution for immunocomplex visualization. The resultant sections were washed, mounted on subbed slides, and covered with slide glasses. Finally, the immunopositive cells were observed under a microscope (Olympus, Tokyo, Japan). Image analysis of immunoreactivity for TH, GFAP, and Iba-1 was conducted using ImageJ software (version 1.54 g, National Institutes of Health, Bethesda, MD, USA). Stereological counting was performed to determine the number of TH^+^ dopaminergic neurons in the substantia nigra. For striatum samples, the number of pixels above the threshold (set to 200) was used to quantify the density of TH^+^ fibers. Microglial and astrocytic activation were evaluated using the same method. The results are presented as a percentage of the threshold area.

#### Western blot analysis

The substantia nigra tissues were homogenized in ice-cold RIPA lysis buffer with 1% protease inhibitor cocktail and centrifuged at 13 000 *×g* and 4 °C for 30 min. The supernatant was collected and protein concentrations were quantified using the BCA protein assay kit (Thermo Fisher Scientific). After loading equal amounts of proteins, each sample was resolved in 12% SDS-polyacrylamide gel. Transblotting of the proteins was performed on polyvinylidene difluoride membranes (Merck Millipore) blocked in Tris-buffered saline with 5% skim milk and 0.1% Tween 20 for 1 h and incubated overnight with primary antibodies against IL-1β (1:2,000; Cell Signaling), TNF-α (1:2,000; GeneTex), and β-actin (1:5,000; AbFrontier) at 4 °C. The membranes were then incubated with horseradish peroxidase-conjugated secondary antibody for 1 h at room temperature. Finally, the membranes were immersed in enhanced chemiluminescence reagents (Thermo Fisher Scientific) and the blots were visualized using a luminescence analyzer (Fusion Solo, Vilber Lourmat, France). The density of each blot was evaluated using GelQuant.Net software.

### Statistical analysis

All data are presented as the mean ± standard deviation (SD) of multiple independent experiments. Differences between two groups were assessed using a one-tailed Student’s *t*-test. For the animal studies, statistical analyses were conducted using GraphPad 9.0 (San Diego, CA, USA) with one-way analysis of variance followed by Tukey’s multiple comparison test. Statistical significance was set at *p* < 0.05.

## Results

### Successful preparation of Lf-ASX-LPs

Firstly, we optimized the ratio of initial ASX and total lipid weight (µg/mg) to maximize the EE and LC based on the appropriate size and zeta potential of LPs. The EE and LC were improved as ASX content increased (Fig. 1B); 2.94 µg/mg ASX showed the maximum EE and LC (89.41 ± 0.40% and 0.139 ± 0.001%, respectively). Consequently, we selected 2.94 µg ASX/mg lipid weight for further experiments, which corresponded to a PDI of 0.13 ± 0.01, particle size of 134.7 ± 0.95 nm, and zeta potential of − 8.40 ± 1.33 mV (Supplementary Fig. S2A and B).

We investigated the optimal composition of lipids to maximize EE and LC by varying the ratios of DPPC and DOPC. A DPPC: DOPC ratio of 1:3 led to the highest EE (97.62 ± 0.28%) and LC (0.248 ± 0.001%, Fig. 1C) and lowest diameter (90.17 ± 0.78 nm), PDI (0.16 ± 0.01, Supplementary Fig. S2C), and zeta potential (–10.92 ± 2.70 mV, Supplementary Fig. S2D). Thus, LPs with a DPPC: DOPC ratio of 1:3 were chosen for further analysis.

We conjugated Lf on the surface of LPs via the maleimide group. Accordingly, we prepared thiolated Lf using Traut’s reagent. The molar ratios between Lf and Traut’s reagent were chosen as 1:40 based on the molar ratio of the thiol group to Lf (Supplementary Fig. S3A). We found a linear increase in the concentration of conjugated Lf as the initial concentration increased (Fig. 1D). The average particle size, PDI, and zeta potential were suitable (Supplementary Fig. S2E and F). We examined the CE using the BCA protein assay (Supplementary Fig. S3B). Based on the results in Fig. 1D and Fig. S3B, the optimal concentration of Lf for subsequent experiments was determined to be 1.5 mg/mL. The CE value of Lf on the surface of LPs was 79.36 ± 0.69% as determined by the ELISA test. While the average particle size, PDI, and surface charge of blank LPs were 93.80 ± 1.11 nm, 0.15 ± 0.01, and − 8.82 ± 1.38 mV, respectively, the average particle size of Lf-ASX-LPs slightly increased to 109.83 ± 1.05 nm, with a corresponding PDI of 0.18 ± 0.01 and zeta potential of − 9.48 ± 1.11 mV (Fig. 1E and F). The average particle size, PDI, and surface charge of Lf-blank LPs were 105.63 ± 1.20 nm, 0.16 ± 0.01, and − 9.28 ± 1.29 mV, respectively. The atomic force microscopy images of Lf-ASX-LPs confirmed that the particles were spherical with a size of ~ 100 nm, consistent with the dynamic light scattering results (Fig. 1G). In addition, we confirmed the sustained release of ASX from Lf-ASX-LPs compared with the ASX suspension (Supplementary Fig. S2G). The stability of Lf-ASX-LPs during storage at 4 °C is also shown in Supplementary Fig. S4A and B. Lf-ASX-LPs were also stable for 3 days at 37 °C (Supplementary Fig. S4C and D). Additionally, we confirmed the enhanced chemical stability of ASX in the liposomal formulation following accelerated stability testing performed at elevated temperatures of 45 °C and 60 °C, as demonstrated in Supplementary Fig. S4E and F, underscoring the protective role of this liposomal platform. The size and zeta potential of LPs used in the Transwell^®^ and biodistribution studies are presented in Supplementary Fig. S5.

### No cytotoxicity associated with ASX and other lipids

The MTT assay confirmed that there were no significant differences in viability between SH-SY5Y, BV-2, and HBMECs at 8 mg/mL lipids and 18 µM ASX compared with the control treatment (Supplementary Fig. S6A–C), confirming the lack of cytotoxicity. We also found no cytotoxicity in SH-SY5Y cells under additional exposure to 1 mM MPP^+^ for 24 h. Based on the viability, we maintained a consistent number of cells throughout the DCF assay, ensuring the accurate measurement of intracellular ROS levels based on fluorescence intensity (Supplementary Fig. S6D). Additionally, Lf-ASX-LPs at ASX concentrations in the range of 5–36 µM showed no significant difference in cell viability over all the concentrations compared to controls (Supplementary Fig. S6E).

### Lf enhanced BBB penetration and cellular uptake

We evaluated the permeation across the endothelial layer and cellular uptake of Lf-functionalized LPs in the Transwell^®^ study with HBMECs and SH-SY5Y cells (Fig. 2A). Lf conjugation promoted the penetration of LPs across the endothelial layer. The fluorescence intensity of Lf-OG488-LPs in the basolateral chamber under the endothelial layer was higher than that of LPs without Lf conjugation after 6 h (*p* < 0.05), 12 h (*p* < 0.01), and 24 h (*p* < 0.05) of incubation (Fig. 2B). In this co-culture model, LPs were internalized into SH-SY5Y cells in a time-dependent manner (Fig. 2C). Compared with OG488-LPs without Lf, Lf-OG488-LP-treated SH-SY5Y cells showed 3- (*p* < 0.001), 7.5- (*p* < 0.01), 14- (*p* < 0.01), and 16.7-fold (*p* < 0.0001) increases in fluorescence intensity after 3, 6, 12, and 24 h of incubation, respectively. After 12 h of incubation, there was no significant difference in the cellular uptake of Lf-OG488-LPs by SH-SY5Y cells (Fig. 2C), reflecting the uptake limit for Lf-conjugated LPs at 12 h after incubation. The results suggested that Lf conjugation facilitated the penetration of LPs across the endothelial layer and enhanced the uptake of LPs into SH-SY5Y cells.

### Cytoprotective activity against MPP^+^-induced cytotoxicity

**Fig. 3:**
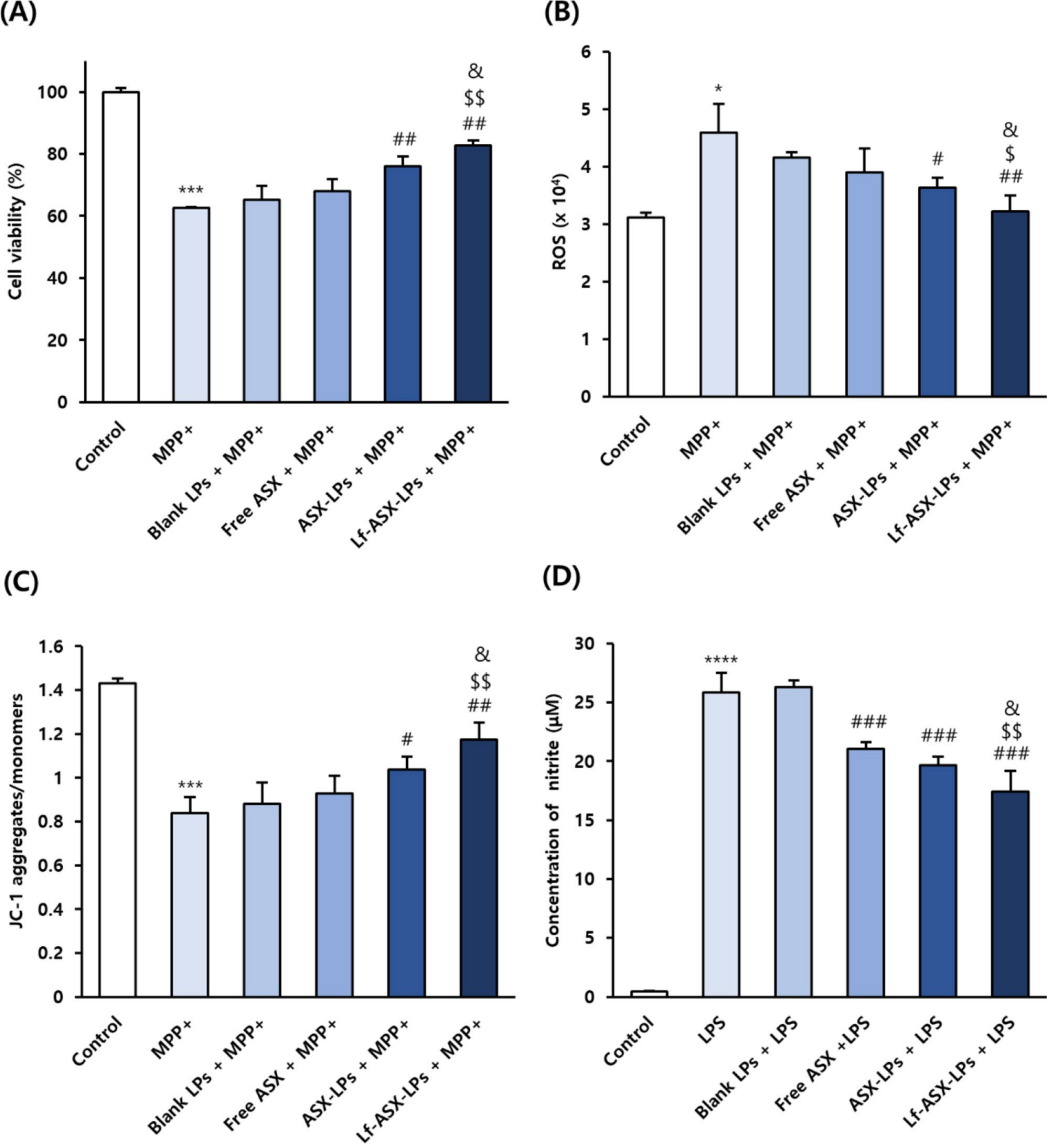
In vitro evaluation of antioxidant performance. (**A**) Viability of SH-SY5Y cells based on MTT assay. Cells were pretreated with blank LPs, free ASX, ASX-LPs, or Lf-ASX-LPs for 6 h before exposure to 2 mM MPP^+^ for 24 h. (**B**) ROS levels determined from DCF fluorescence intensity. After pre-treatment with the indicated agents for 6 h, SH-SY5Y cells were exposed to 1 mM MPP^+^ for 24 h. (**C**) JC-1 aggregate: monomer ratio in SH-SY5Y cells. After pre-treatment with the indicated agents for 6 h, SH-SY5Y cells were incubated with 2 mM MPP^+^ for another 24 h. (**D**) Nitrite concentrations based on Griess assay. After pre-treatment with the agents for 6 h, BV-2 cells were incubated with 500 ng/mL LPS for another 24 h. All data are presented as the mean ± SD (*n* = 3). *p < 0.05, ***p < 0.001, ****p < 0.0001 vs. control; ^#^*p* < 0.05, ^##^*p* < 0.01, ^###^*p* < 0.001 vs. MPP^+^ or LPS; ^$^*p* < 0.05, ^$$^*p* < 0.01 vs. free ASX + MPP^+^ or free ASX + LPS; ^&^*p* < 0.05 vs. ASX-LPs + MPP^+^ or ASX-LPs + LPS

We measured the viability of SH-SY5Y cells after MPP^+^ exposure to evaluate the protective role of Lf-ASX-LPs (Fig. 3A). In this experiment, the untreated and MPP^+^-only treatment groups served as negative and positive controls, respectively. ASX-LPs and Lf-ASX-LPs ameliorated cell loss by 13.5% and 20.1%, respectively (*p* < 0.01), compared with that under MPP^+^-induced cytotoxicity, whereas blank LPs and free ASX had no discernible effect. The Lf-ASX-LP pretreatment was associated with increased cell viability compared with the ASX-LP (*p* < 0.05) and free ASX treatments (*p* < 0.01). These results demonstrated that the liposomal ASX-based formulation and Lf functionalization exerted cytoprotective effects against MPP^+^*-*induced cytotoxicity in SH-SY5Y cells.

### Intracellular antioxidant activity against MPP^+^-induced cytotoxicity

We further investigated the protective role of Lf-ASX-LPs against MPP^+^-induced cytotoxicity based on the levels of intracellular ROS measured using the DCFH-DA fluorescent probe (Fig. 3B). The 1 mM MPP^+^ treatment increased the production of intracellular ROS (*p* < 0.05). The pretreatment with ASX-LPs and Lf-ASX-LPs resulted in 21.0% (*p* < 0.05) and 30.0% (*p* < 0.01) decreases in DCF fluorescence intensity, respectively, compared to the MPP^+^-only treatment. Similarly, incubation with ASX-LPs or Lf-ASX-LPs reduced the intracellular ROS levels compared with the blank LP treatment (*p* < 0.01). More importantly, there was no difference in fluorescence intensity between the Lf-ASX-LPs and control groups, indicating the potent antioxidant capacity of the formulation.

### Ameliorated reduction of MMP under MPP^+^-induced cytotoxicity

We evaluated the changes in MMP levels after exposure to MPP^+^ using JC-1 fluorescent probes and found that 2 mM MPP^+^ resulted in a 41.4% decrease (*p* < 0.001) in the ratio of JC-1 aggregates: monomers in SH-SY5Y cells (Fig. 3C). The cells pretreated with ASX-LPs and Lf-ASX-LPs followed by MPP^+^ exposure showed 23.7% (*p* < 0.05) and 40.1% (*p* < 0.01) increases in the aggregate: monomer ratios compared with cells under MPP^+^ exposure only. ASX-LP- and Lf-ASX-LP-treated cells showed 17.5% (*p* < 0.05) and 33.1% (*p* < 0.01) increases in the aggregate: monomer ratios compared with the blank LP-treated cells. More importantly, Lf-ASX-LP-treated cells showed 26.5% and 13.3% increases in MMP levels compared with free ASX- (*p* < 0.01) and ASX-LP-treated cells (*p* < 0.05).

### Decreased extracellular NO levels under LPS-induced cytotoxicity

We indirectly measured extracellular NO levels using the Griess assay (Fig. 3D). LPS-treated BV-2 cells showed 57-times higher levels of nitrite (*p* < 0.0001) compared with the untreated cells. Pretreatment with free ASX, ASX-LPs, and Lf-ASX-LPs decreased the extracellular LPS-induced nitrite levels compared with the LPS-only treatment (*p* < 0.001). The nitrite levels of groups treated with ASX-containing formulations were also lower than those in the blank LP-treated group (*p* < 0.001 for all three groups). Moreover, the Lf-ASX-LP-treated group exhibited decreased nitrite concentrations compared with the free ASX- (*p* < 0.01) and ASX-LP-treated groups (*p* < 0.05). The Lf-ASX-LP treatment facilitated the most effective reduction in nitrite levels (~ 32%) compared with the LPS treatment.

### Enhanced penetration of Lf-conjugated LPs into the brain

**Fig. 4:**
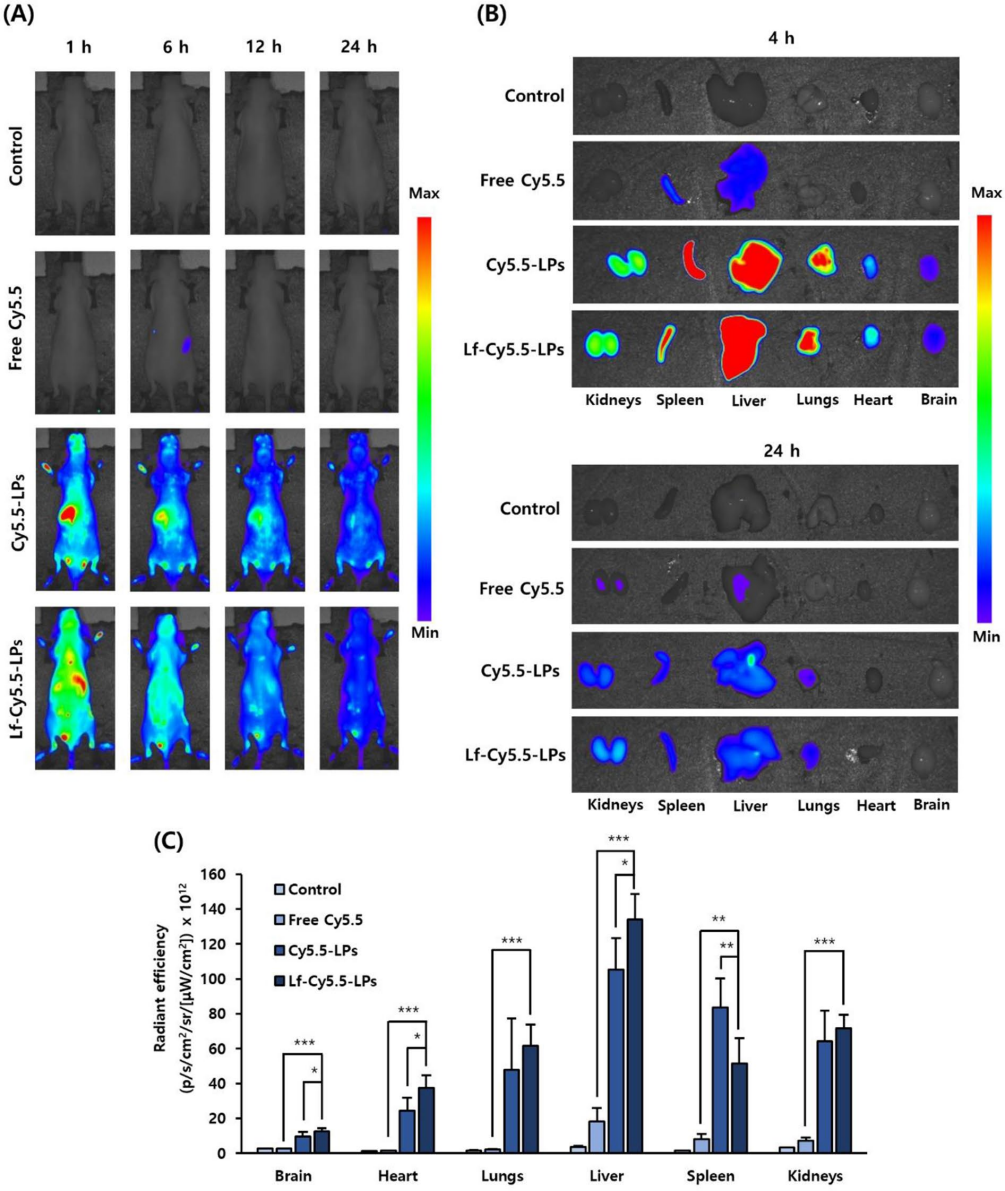
Biodistribution of LPs in BALB/c nude mice. (**A**) Real-time fluorescence imaging of control, free Cy5.5, Cy5.5-LPs, and Lf-Cy5.5-LPs groups at various time points post-injection. (**B**) Accumulation of Cy5.5 fluorescent probe in the brain and other organs at 4 and 24 h after injection. (**C**) Quantification of fluorescence intensity in the brain and other organs at 4 h after injection. Data are presented as the mean ± SD (*n* = 5). *p < 0.05, **p < 0.01, ***p < 0.001

We observed a greater accumulation of Lf-Cy5.5-LPs than free Cy5.5 or Cy5.5-LPs in rodent brains (Fig. 4A). The Lf-conjugated LPs had the highest accumulation in the brain at 1 h post-injection. After 24 h, almost all LPs were removed from the brains in the Cy5.5-LP- and Lf-Cy5.5-LP-treated groups. After intravenous injection, free Cy5.5 was immediately eliminated from circulation. At 4 h and 24 h post-injection, the mice were sacrificed, and their brains and other major organs were isolated for ex vivo imaging. The fluorescence intensity of Cy5.5 was higher in the Lf-Cy5.5-LP-treated group at 4 h than in the free-Cy5.5- and Cy5.5-LP-treated groups (*p* < 0.001 and *p* < 0.05, respectively; Fig. 4B and C), confirming that Lf conjugation facilitated the BBB penetration of LPs. The ex vivo images at 24 h indicated that the majority of LPs were eliminated within 1 d after treatment. The remaining LPs were mainly distributed in the liver, kidneys, spleen, and lungs.

### Ameliorated behavioral impairment in the MPTP-induced PD model

**Fig. 5:**
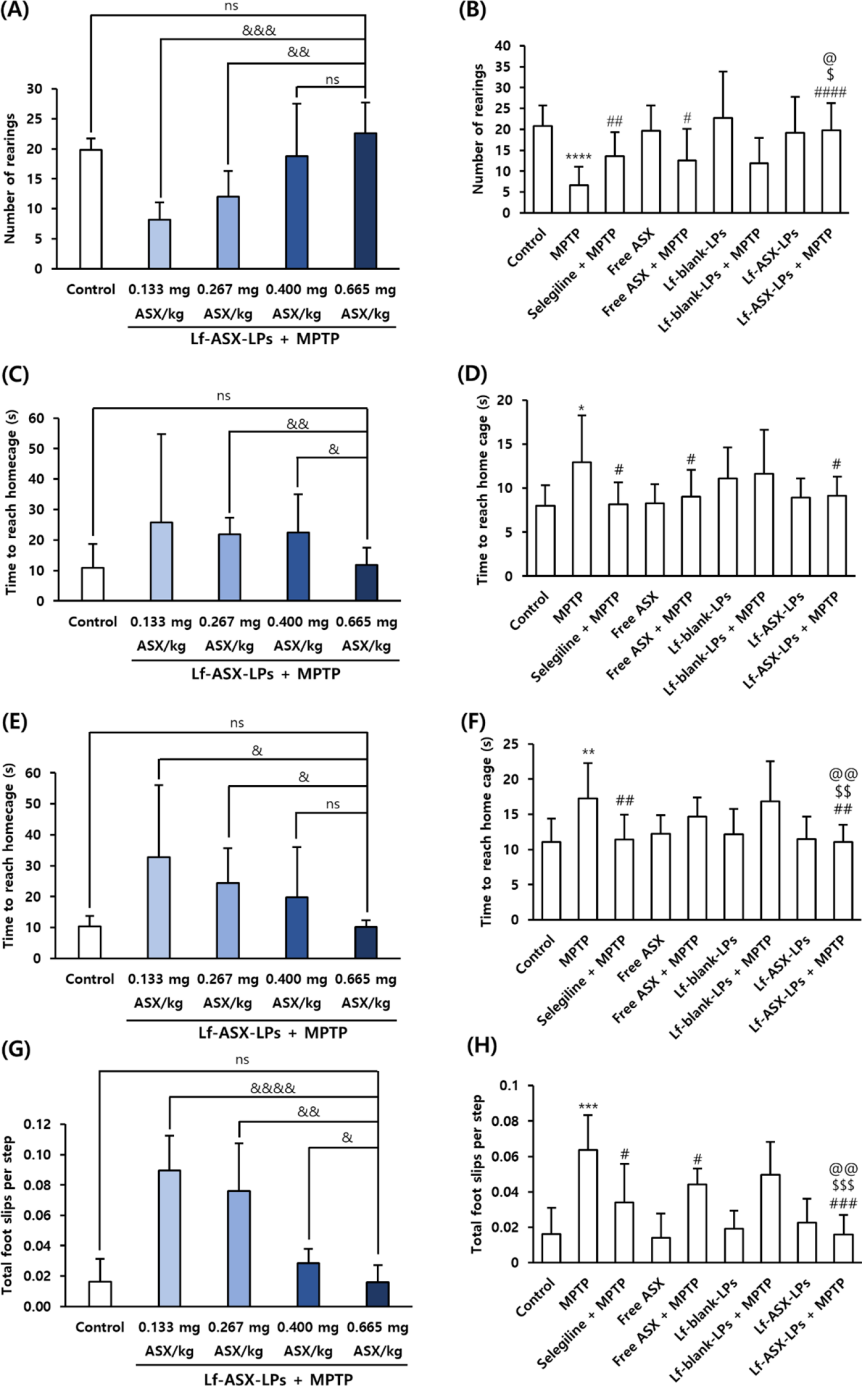
Behavioral evaluation in MPTP-treated model. (**A**, **B**) Number of rearings recorded over 3 min. The time to reach the home cage was determined from the (**C**, **D**) beam walking and (**E**, **F**) challenging walking experiments. (**G**, **H**) Number of foot slips per step during the challenging walking experiment. All data are presented as the mean ± SD (*n* = 9). *p < 0.05, **p < 0.01, ***p < 0.001, ****p < 0.0001 vs. control; ^#^*p* < 0.05, ^##^*p* < 0.01, ^###^*p* < 0.001, ^####^*p* < 0.0001 vs. MPTP; ^$^*p* < 0.05, ^$$^*p* < 0.01, ^$$$^*p* < 0.001 vs. free ASX + MPTP; ^@^*p* < 0.05, ^@@^*p* < 0.01 vs. Lf-blank-LPs + MPTP; ^&^*p* < 0.05, ^&&^*p* < 0.01, ^&&&^*p* < 0.001, ^&&&&^*p* < 0.0001; ns: non-significant

In the cylinder test, MPTP injection resulted in a decrease in the number of rearings in comparison to the control, indicating impairment in the exploring activity of mice. Treatment with Lf-ASX-LPs induced a dose-dependent enhancement in the rearing behavior (Fig. 5A). Importantly, the highest dose of Lf-ASX-LPs recovered the exploring activity to a similar level to that of the control. The comparison study showed that Lf-ASX-LPs significantly restored behavior (*p* < 0.0001) (Fig. 5B).

The beam walking test consistently displayed that Lf-ASX-LPs treatment dose-dependently alleviated motor dysfunction mediated by MPTP (Fig. 5C). The MPTP group required more time to reach home compared with the control (*p* < 0.05) and Lf-ASX-LPs significantly improved motor function in the beam walking test (*p* < 0.05, compared with MPTP only; Fig. 5D). However, there was no difference between ASX and Lf-ASX-LPs treatment groups.

Similar results were obtained in the challenging beam walking test, as Lf-ASX-LPs dose-dependently decreased time to reach the home cage in comparison to MPTP (Fig. 5E). In a comparison study, Lf-ASX-LPs showed a significant improvement in motor function compared with MPTP injection (*p* < 0.01, compared with MPTP; Fig. 5F). Importantly, there was a significant difference between free ASX + MPTP and Lf-ASX-LPs + MPTP, indicating the enhanced neuroprotective effect of the liposomal formulation (*p* < 0.01, Fig. 5F).

We also observed dose-dependent decreases in the number of foot slips per step in the challenging beam tests (Fig. 5G). In a comparison study, Lf-ASX-LPs showed significant efficacy in reducing foot slips (*p* < 0.001). Notably, there was a remarkable difference between free ASX + MPTP and Lf-ASX-LPs + MPTP (*p* < 0.001, Fig. 5H).

### Mitigated MPTP-induced DA reductions in the MPTP-induced PD model

**Fig. 6:**
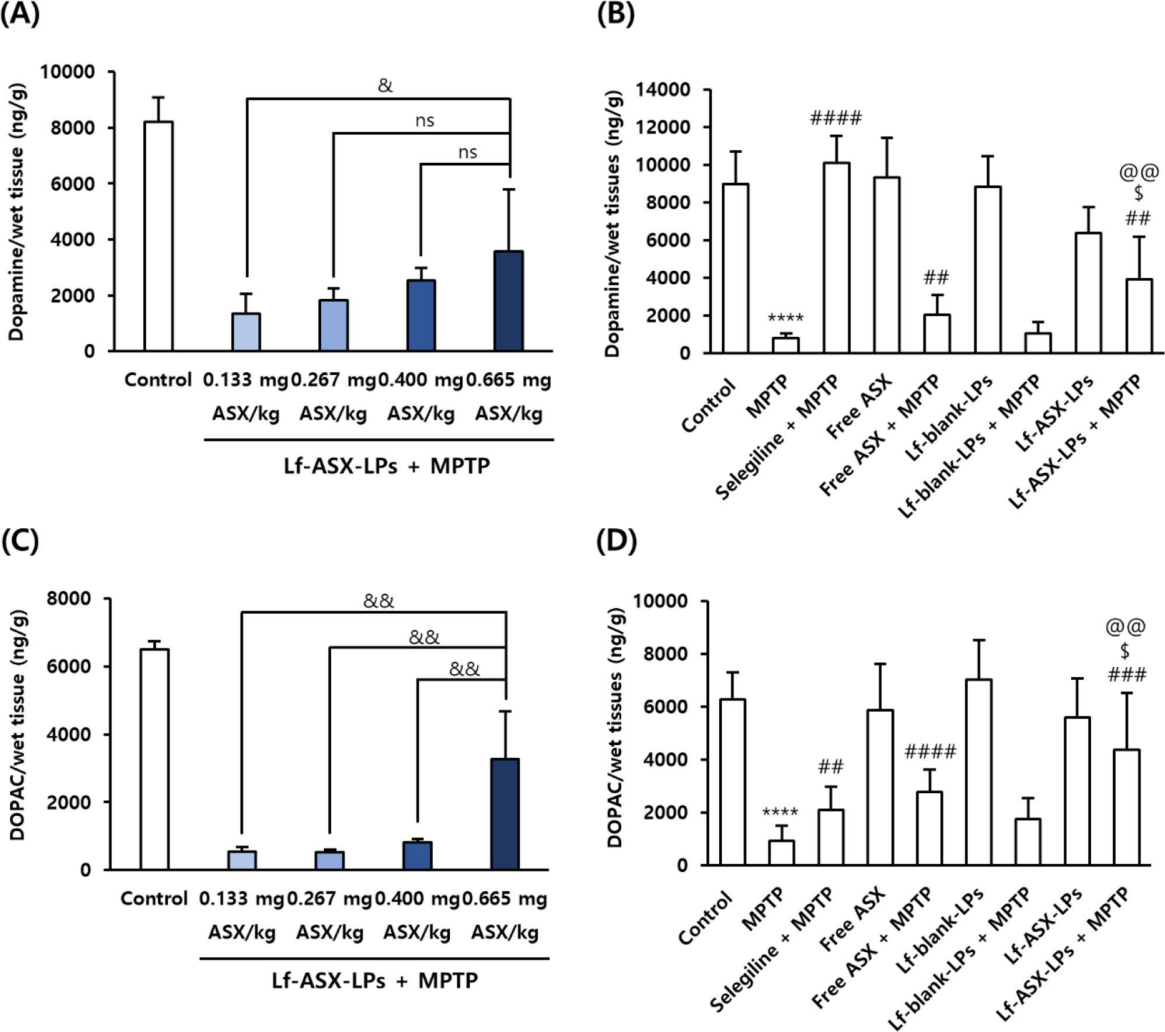
DA and DOPAC quantification in MPTP-treated model. HPLC estimates for (**A**, **B**) DA and (**C**, **D**) DOPAC concentrations in the striatum after treatment with different formulations. All data are presented as the mean ± SD (*n* = 9). ****p < 0.0001 vs. control; ^##^*p* < 0.01, ^###^*p* < 0.001, ^####^*p* < 0.0001 vs. MPTP; ^$^*p* < 0.05 vs. free ASX + MPTP; ^@@^*p* < 0.01 vs. Lf-blank-LPs + MPTP; ^&^*p* < 0.05, ^&&^*p* < 0.01; ns: non-significant

Following HPLC analysis of neurochemicals, we observed that MPTP significantly reduced the levels of DA and DOPAC, which were dose-dependently restored by Lf-ASX-LPs (Fig. 6A and C). In a comparison study, MPTP treatment demonstrated a significant decrease in DA levels compared with the control (*p* < 0.0001) and Lf-ASX-LPs showed significantly ameliorated DA depletion induced by MPTP (*p* < 0.01). The restorative effect of Lf-ASX-LPs was higher than that of free ASX (Fig. 6B). Similar to DA, DOPAC levels in the MPTP-treated mice were significantly lower than those in the control (*p* < 0.0001). Lf-ASX-LPs significantly attenuated DOPAC loss induced by MPTP (*p* < 0.001) (Fig. 6D). Notably, there was a significant difference between free ASX + MPTP and Lf-ASX-LPs + MPTP (*p* < 0.05), indicating superior activity of the liposomal formulation.

### Attenuation of MPTP-induced dopaminergic neuronal damage

**Fig. 7:**
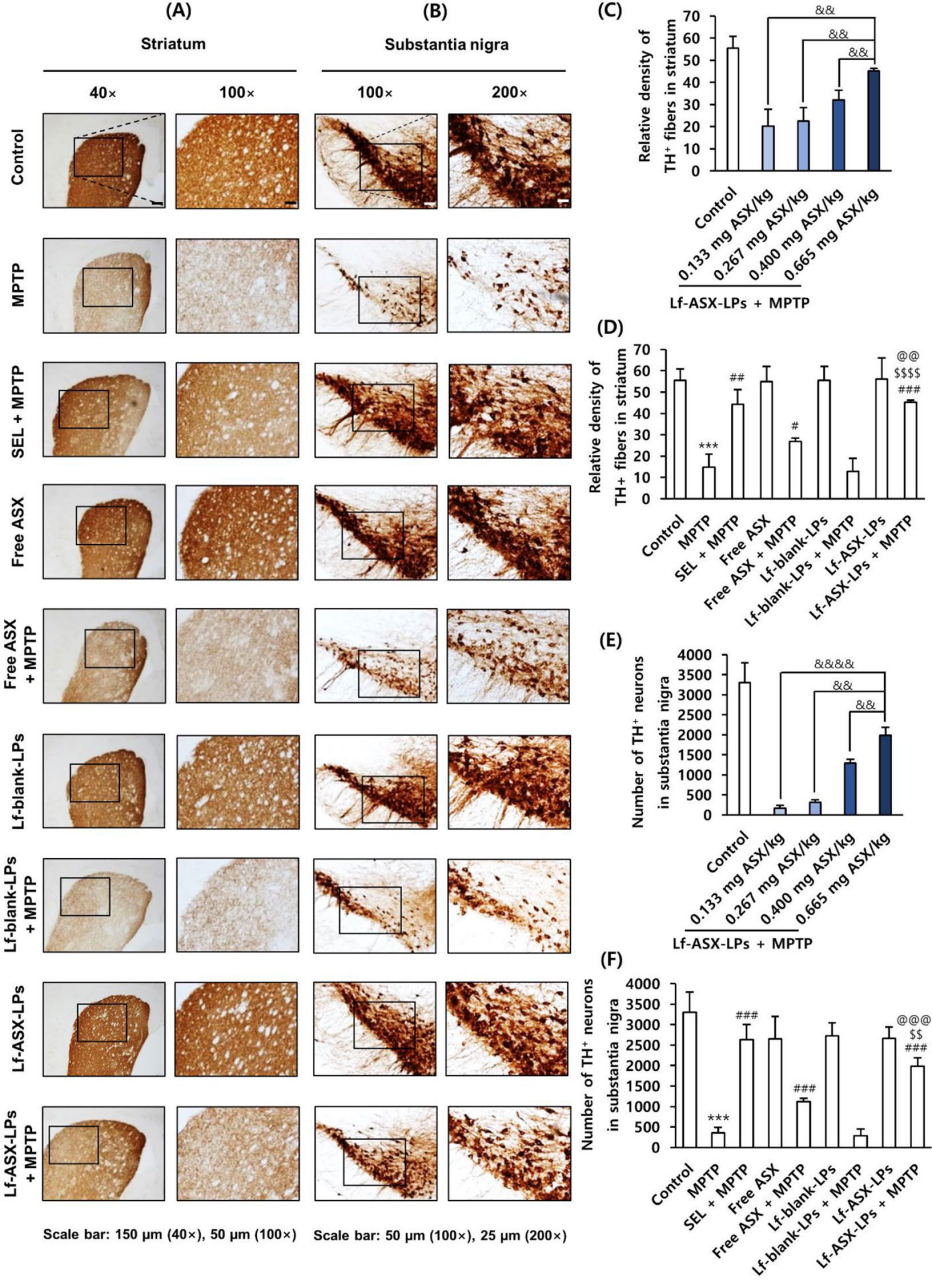
Protective effects of different formulations in dopaminergic neurons under MPTP-induced neurotoxicity. (**A**) Expression of TH^+^ fibers in striatum. (**B**, **C**) Quantification of TH^+^ dopaminergic fibers in striatum. (**D**) Expression of TH^+^ dopaminergic neurons in substantia nigra. (**E**, **F**) Quantification of TH^+^ dopaminergic neurons in substantia nigra. All data are presented as the mean ± SD (*n* = 3). ***p < 0.001 vs. control, ^#^*p* < 0.05, ^##^*p* < 0.01, ^###^*p* < 0.001 vs. MPTP, ^$$^*p* < 0.01, ^$$$$^*p* < 0.0001 vs. free ASX + MPTP; ^@@^*p* < 0.01, ^@@@^*p* < 0.001 vs. Lf-blank-LPs + MPTP; ^&&^*p* < 0.01, ^&&&&^*p* < 0.0001

IHC staining results for TH^+^ neurons showed that MPTP caused the loss of dopaminergic neurons in the nigrostriatal pathway (Fig. 7A-F and Supplementary Fig. S7). Injection with Lf-ASX-LPs significantly and dose-dependently protected the neurons from MPTP-induced neuronal loss in the striatum (Fig. 7A-C) and the substantia nigra (Fig. 7D-F). The quantitative results of dopaminergic fibers in the striatum indicated that Lf-ASX-LPs treatment spared the fibers from MPTP-induced injury (*p* < 0.001), which was more evident than that from free ASX (*p* < 0.0001) (Fig. 7C). The number of TH^+^ dopaminergic neurons within the substantia nigra was significantly reduced by the MPTP treatment. Lf-ASX-LPs exhibited neuroprotective effects against MPTP toxicity as the loss of TH^+^ dopaminergic neurons was significantly and dose-dependently attenuated by our formulation (Fig. 7D-F). As shown in Fig. 7F, the protective effect of Lf-ASX-LPs was more evident than that of free ASX (*p* < 0.01), implying superior neuroprotective activity.

### Anti-neuroinflammatory effect in the MPTP-treated brains

**Fig. 8:**
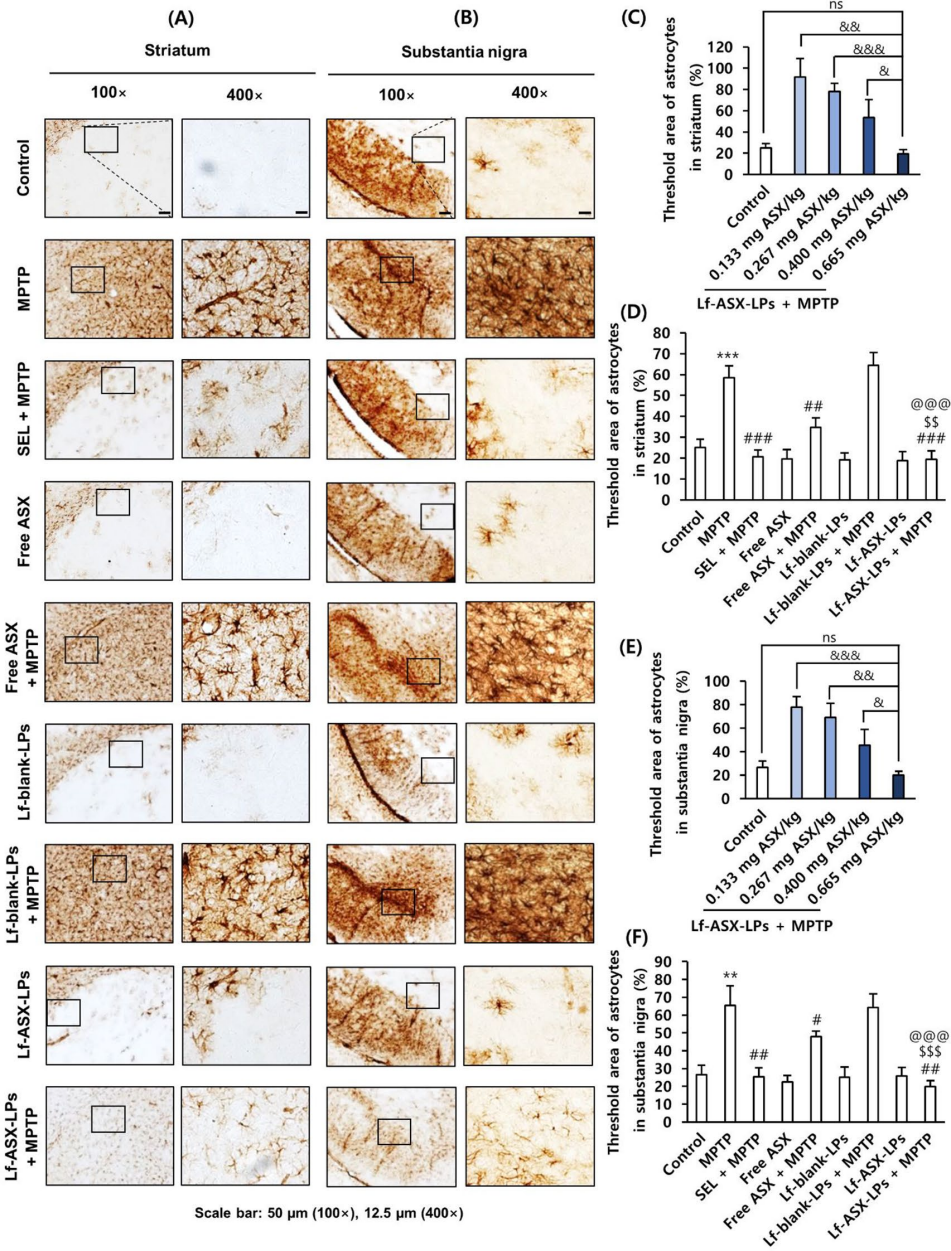
Astrocyte activation in MPTP-induced toxicity model. (**A**) Activated GFAP-positive astrocytes in striatum. (**B**, **C**) Quantification of activated GFAP-positive astrocytes in striatum. (**D**) Activated GFAP-positive astrocytes in substantia nigra. (**E**, **F**) Quantification of activated GFAP-positive astrocytes in substantia nigra. All data are presented as the mean ± SD (*n* = 3). **p < 0.01, ***p < 0.001 vs. control, ^#^*p* < 0.05, ^##^*p* < 0.01, ^###^*p* < 0.001 vs. MPTP, ^$$^*p* < 0.01, ^$$$^*p* < 0.001 vs. free ASX + MPTP; ^@@@^*p* < 0.001 vs. Lf-blank-LPs + MPTP; ^&^*p* < 0.05, ^&&^*p* < 0.01, ^&&&^*p* < 0.001; ns: non-significant

**Fig. 9:**
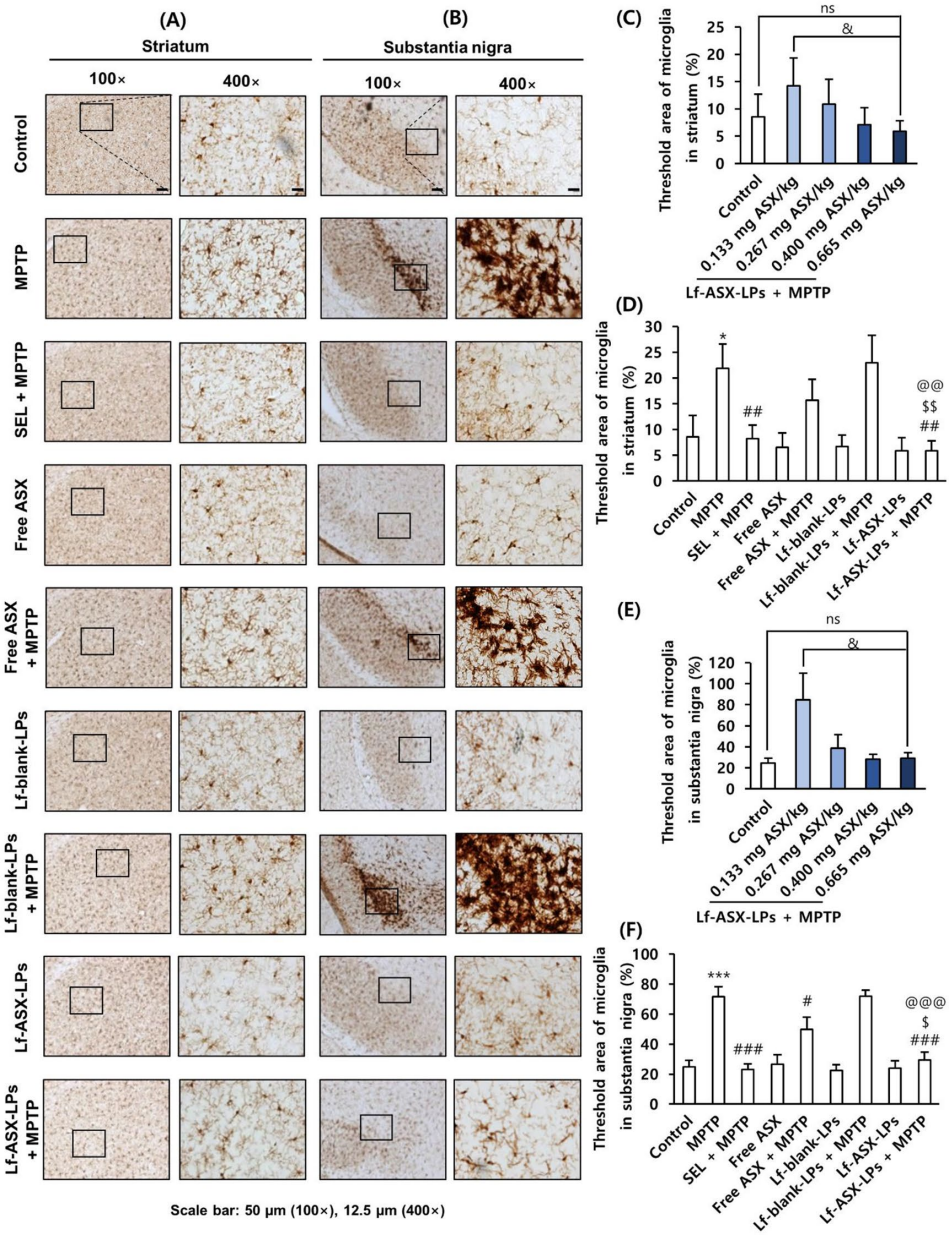
Microglial activation in MPTP-induced toxicity model. (**A**) Immunostaining of Iba-1 in striatum. (**B**, **C**) Quantification of activated Iba-1-positive microglia in striatum. (**D**) Immunostaining of Iba-1 in substantia nigra. (**E**, **F**) Quantification of activated Iba-1-positive microglia in substantia nigra. All data are presented as the mean ± SD (*n* = 3). *p < 0.05, ***p < 0.001 vs. control, ^#^*p* < 0.05, ^##^*p* < 0.01, ^###^*p* < 0.001 vs. MPTP, ^$^*p* < 0.05, ^$$^*p* < 0.01 vs. free ASX + MPTP; ^@@^*p* < 0.01, ^@@@^*p* < 0.001 vs. Lf-blank-LPs + MPTP; ^&^*p* < 0.05; ns: non-significant

As shown in Figs. 8 and 9, MPTP injection mediated neuroinflammatory responses as determined by IHC for GFAP, a marker for astrocytes, and Iba-1, a marker for microglia, and western blots for IL-1β and TNF-α. MPTP injection significantly increased the number of activated astrocytes in the striatum (Fig. 8A-C) and substantia nigra (Fig. 8D-F). In addition, injection of the neurotoxicant significantly increased the number of activated microglia cells in the striatum (Fig. 9A-C) and substantia nigra (Fig. 9D-F).

Importantly, Lf-ASX-LPs significantly and dose-dependently attenuated glial activation in the striatum and substantia nigra, as assessed by the density of IHC staining and the counting of activated glial cells (Supplementary Fig. S8 and Fig. S9). Moreover, the attenuating effects of Lf-ASX-LPs were more significant than those of free ASX both in the striatum and substantia nigra.

**Fig. 10:**
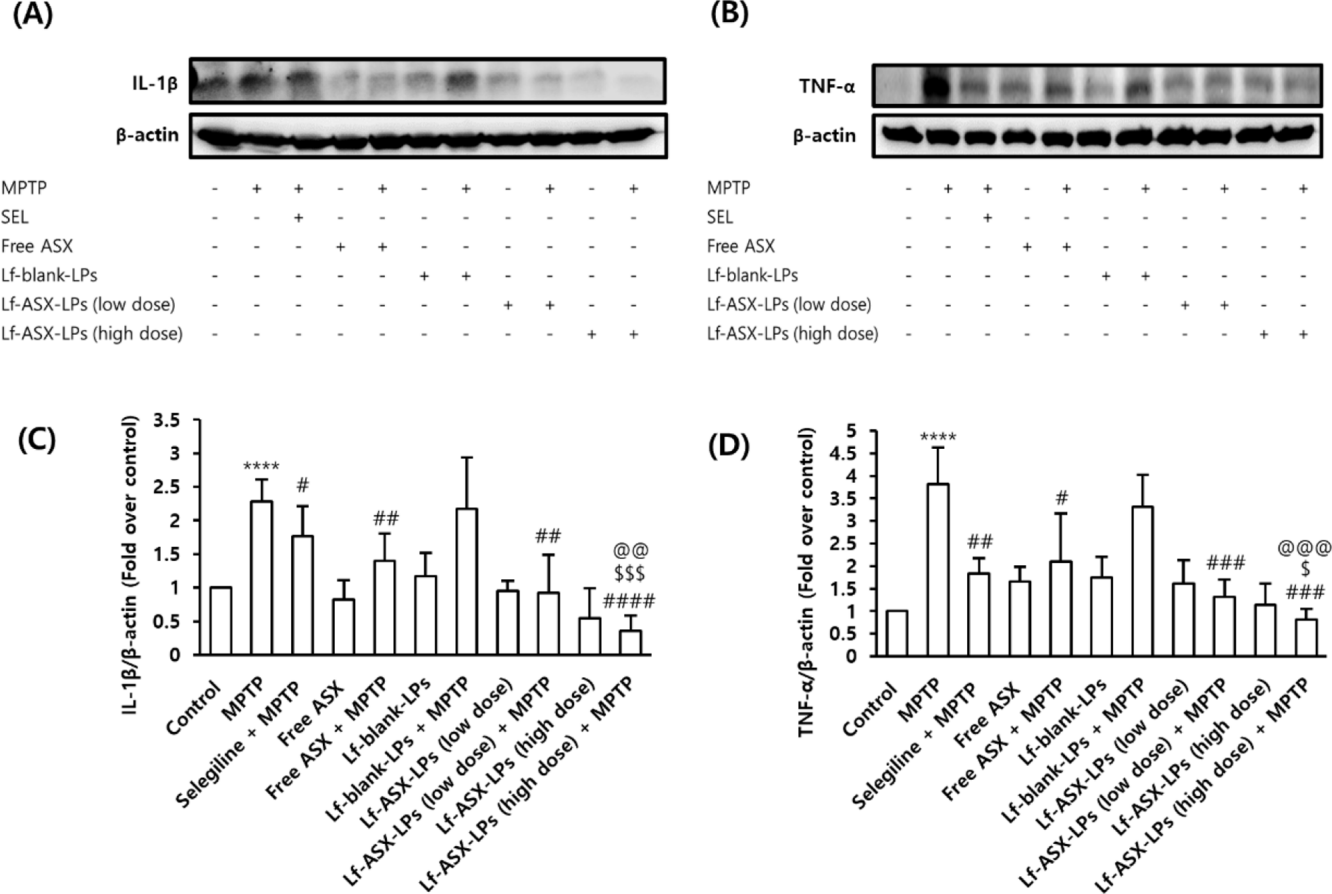
Western blot analysis of proinflammatory cytokines within substantia nigra. Qualitative analysis of (**A**) IL-1β and (**B**) TNF-α. Quantitative analysis of (**C**) IL-1β and (**D**) TNF-α. All data are presented as the mean ± SD (*n* = 6). Low dose = 0.400 mg ASX/kg and high dose = 0.665 mg ASX/kg. ****p < 0.0001 vs. control; ^#^*p* < 0.05, ^##^*p* < 0.01, ^###^*p* < 0.001, ^####^*p* < 0.0001 vs. MPTP; ^$^*p* < 0.05, ^$$$^*p* < 0.001 vs. free ASX + MPTP; ^@@^*p* < 0.01, ^@@@^*p* < 0.001 vs. Lf-blank-LPs + MPTP

Finally, we performed western blotting for IL-1β and TNF-α levels in the substantia nigra. The results indicated that the release of cytokines was significantly increased by MPTP injection (Fig. 10A-D). Lf-ASX-LPs treatment alleviated the expression of proinflammatory cytokines (Fig. 10A-B). Full blots of IL-1β and TNF-α were added in Supplementary Fig. S10. Importantly, the alleviating effects of Lf-ASX-LPs were significantly higher than those of free ASX, suggesting that the formulation enhanced the anti-inflammatory effect of ASX.

## Discussion

In this study, we successfully formulated Lf-ASX-LPs and confirmed their antioxidative effects against MPP^+^-induced cytotoxicity in SH-SY5Y cells. The Lf conjugation enhanced the penetration of LPs into the brain in vivo, resulting in notable neuroprotection in the MPTP-induced PD mouse model. These findings highlight the potential of Lf-ASX-LPs as a neuroprotective therapeutic agent against PD.

LPs are small spherical particles composed of lipids. Natural and synthetic phospholipids form a lipid bilayer for encapsulating hydrophobic therapeutic agents along with an aqueous core for hydrophilic drug loading. In this study, the encapsulation of ASX, a super hydrophobic compound (logP = 10.3), in the bilayered LPs increased its solubility and enhanced permeation into target tissues. Encapsulation of ASX in a nanocarrier increases its solubility and stability, in turn promoting its bioavailability [33–35]. Our LP-based formulations had an ASX concentration of 200 µg/mL, whereas the solubility of free ASX in ethanol and water is 38 and 8 × 10^− 10^ µg/mL, respectively [36, 37]. Gu et al. [33] found that PEGylated ASX-loaded LPs improved the aqueous solubility of ASX and ameliorated cognitive disorder by reducing formaldehyde-induced β-amyloid aggregation.

Assessing the release profile of hydrophobic drugs like ASX (logP = 10.3) from LPs could be challenging. To maintain sink conditions during the in vitro release study, we used a release medium containing 0.5% Tween 80. However, after 24 h, the cumulative release of encapsulated ASX was only 8%, indicating good integration of LNPs in physiological conditions (Supplementary Fig. S2G). A similar release pattern was also observed in an in vitro release study of curcumin-loaded PEGylated magnetic liposomes [38]. We postulated that the slow release was attributed to the sustained release of the hydrophobic drug, *via* diffusion, from the lipid bilayer into aqueous solution.

In terms of lipid composition, LPs formulated with saturated phospholipids, such as DPPC, provide a stable and rigid bilayer structure that prevents drug leakage. In contrast, unsaturated phospholipids such as DOPC lend flexibility, which enhances the permeability of LPs. Thus, the combination of saturated and unsaturated phospholipids in liposome formulations allows for the optimization of stability and flexibility, among other clinically relevant features. Cholesterol provides structural stability in eukaryotic cell membranes. In liposomal preparation, it is usually used at a ratio of < 30% of the total lipids to enhance the rigidity and stability of the lipid membrane. Here, we used 27% (mole) cholesterol.

The BBB is responsible for managing the influx and efflux of biological molecules that are necessary for brain function. While the BBB protects the CNS from harmful neurotoxins, it can also hinder the delivery of therapeutic agents into the brain. To enhance BBB penetration, we conjugated Lf via a thiol-maleimide click reaction using Traut’s reagent and incubated with LPs expressing maleimide-functionalized polyethylene glycol 2000 on their outer surfaces. The concentration of Traut’s reagent determined the degree of thiolization. Thus, we optimized the Lf-to-Traut’s reagent ratio to promote the thiol-maleimide reaction and ensure good bioactivity (Supplementary Fig. S3A). Despite the structural similarities between Lf and Tf, they have distinct receptor binding properties. Moreover, Lf demonstrates a higher brain uptake capacity than Tf, suggesting that Lf is a candidate ligand for drug delivery to the brain [27, 39]. Lalani et al. demonstrated that Lf-functionalized tramadol-loaded nanoparticles exhibited a predominantly antinociceptive effect compared to those functionalized with Tf [39]. LfR is expressed on brain endothelial cells and neurons, and is responsible for transporting Lf across the BBB into the brain [23, 40]. Notably, Faucheux et al. showed that LfR expression was upregulated in the nigral dopaminergic neurons of patients with PD [41].

Eigenmann et al. found that HBMECs were the most appropriate cell line for modeling BBB penetration in vitro compared with other immortalized human brain capillary endothelial cell lines due to their barrier tightness and paracellular permeability [42]. They also indicated that cell seeding density and incubation time played critical roles in transendothelial electrical resistance (TEER). The highest TEER value was observed in HBMECs cultivated at a density of 4.5 × 10^4^ for 3.19 d. Surprisingly, the BBB model using HBMECs in co-cultures with human pericytes and astrocytes showed poorer TEER compared with HBMEC monocultures, which could be due to the difference in culture conditions and cell types. In this study, we applied a density of 5 × 10^4^ HBMECs cultured for 3 d in the Transwell^®^ assay, while SH-SY5Y cell cultures were performed at a cell density of 1 × 10^5^ overnight. Lf-OG488-LP treatment increased the fluorescence intensity in SH-SY5Y cells in a time-dependent manner compared with OG488-LPs, indicating increased BBB permeability (Fig. 2C).

Mitochondrial dysfunction is a key driver in the pathogenesis of PD [43, 44]. Mitochondrial dysregulation results in oxidative stress, leading to dopaminergic neuron degeneration. Emerging evidence in animal models supports the crucial role of inflammation-induced neurodegeneration in PD via the activation of microglia and other immune cells in the brain, along with the secretion of proinflammatory cytokines [45, 46]. Neuroinflammation and mitochondrial impairment may participate in the etiology of PD [47, 48]. Proinflammatory cytokines can disrupt mitochondrial function by affecting oxidative phosphorylation and inducing oxidative stress. In turn, mitochondrial dysfunction may lead to immune cell activation and promote neuroinflammation. ASX can modulate neuroinflammation by alleviating oxidative stress and neuroinflammatory factor production [49, 50]. ASX regulates the cellular enzymatic system, including nuclear factor erythroid 2-related factor, to counteract excessive ROS production [51, 52]. The potential of ASX to reduce ROS production may be linked to its restoration of glutathione peroxidase and superoxide dismutase activities. ASX exerted its antioxidant effects in MPP^+^-treated PC12 cells through the HO-1/NOX2 and Sp1/NR1 pathways and its anti-apoptotic effects in 6-hydroxydopamine-induced SH-SY5Y cells via a mitochondria-targeted mechanism [53, 54]. Thus, ASX is a promising agent for the suppression of PD-associated neuroinflammation.

In PD research, MPTP is a strong neurotoxin that causes selective dopaminergic neuron degeneration in the nigrostriatal region in rodent and primate models. After penetrating through the BBB into the brain, MPTP is converted into MPP^+^ by astrocyte-derived monoamine oxidase B. MPP^+^ accumulation in mitochondria of the dopaminergic neurons results in mitochondrial dysfunction. In SH-SY5Y cells, ASX reportedly inhibited MPP^+^-induced ROS production and apoptosis; specifically, 50 µM ASX alleviated oxidative damage by protecting antioxidant enzymes, including superoxide dismutase and catalase, from MPP^+^ [55]. ASX (50 µM) also regulated anti- and pro-apoptotic protein levels via the upregulation of Bax and downregulation of Bcl-2 levels under MPP^+^-induced toxicity [55]. Our in vitro study demonstrated that a lower dose of ASX (18 µM) was sufficient to achieve protective effects against MPP⁺-induced toxicity.

Several studies have demonstrated that reactive nitrogen species, specifically NO, are a key factor in neurodegeneration and the pathogenesis of PD [56]. Although NO is an important regulator of various cellular functions under physiological conditions, the abnormal production of NO causes mitochondrial impairment by inhibiting enzymes of the mitochondrial electron transport chain, eventually leading to neuronal cell death in PD. In this study, we stimulated the production of NO using LPS in microglial BV-2 cells pretreated with free ASX, ASX-LPs, and Lf-ASX-LPs. The free ASX and ASX-LP treatments significantly mitigated NO generation in vitro. Rawat et al. demonstrated that the glycolytic protein glyceraldehyde-3-phosphate dehydrogenase acts as a receptor for Lf in murine macrophages [57]. This suggested that Lf-ASX-LPs exhibited greater attenuation of NO levels compared to ASX-LPs due to their improved cellular uptake ability. These findings confirmed the neuroprotective effect of ASX against LPS-induced NO and the potential of Lf conjugation for maintaining bioactivity in vivo.

Under physiological conditions, DA and other neurotransmitters coordinate the millions of neuronal and muscle cells that control movement. A decline in DA levels disrupts the nigrostriatal pathway, leading to abnormal movement—a hallmark of PD. TH is the primary enzyme regulating DA biosynthesis. Hence, quantification of TH-positive cells is essential for evaluating the neuroprotective effects in PD models. We found that the number of TH-positive neurons was markedly reduced in MPTP-treated mice, while Lf-ASX-LPs ameliorated this loss. Consistent with these results, Wang et al. found that esterified ASX strongly induced the mRNA expression of TH and mitigated the loss of dopaminergic neurons in an MPTP-induced PD model [58]. ASX also enhanced the levels of antioxidant enzymes and suppressed the level of malondialdehyde—a lipid peroxidation marker. In terms of apoptotic cell death, ASX reduced the ratios of Bax/Bcl-2 and cleaved-caspase 3/caspase 3 [58]. Moreover, ASX enhanced the neuroprotective effect against MPTP toxicity via a miR-7/α-synuclein axis [59]. In our study, mice intravenously injected with our formulation (0.665 mg/kg ASX, every other day for 2 weeks) showed significant recovery of TH-positive dopaminergic neurons. At a much higher dose, Lee et al. found that intraperitoneal injection with 30 mg/kg free ASX every day for 28 d increased the number of TH-positive neurons in an MPTP-induced mouse model [55]. Therefore, our formulation demonstrated superior recovery against DA decline and TH impairment compared with other nanoparticle-based formulations utilized in MPTP-induced mouse models [32, 60]. Katila et al. found that resveratrol-loaded poly(lactic-co-glycolic acid) nanoparticles mitigated DA impairment under MPTP-induced toxicity. Despite showing a 2.1-fold increase in DA concentration compared with the MPTP-treated group, the recovery rate was only 21.7% compared to the control group [32]. In a similar mouse model, our LP-based formulation resulted in a 5.0-fold increase in DA levels compared with MPTP treatment alone, with a recovery rate of 43.5% compared to the control group. A study using retinoic acid-loaded polymeric nanoparticles reported a ~ 2-fold increase in TH^+^ cell numbers in the striatum compared with an MPTP-treated group [60]. Within the same model, our Lf-ASX-LPs showed > 3-fold enhancement in striatal TH^+^ neuron levels compared with MPTP only.

Sustained neuroinflammatory responses strongly affect the progression of PD. Excessive glial cell activation, which induces extensive proinflammatory cytokine release, contributes to the development of neurodegenerative diseases [46]. Iba-1 is considered a pan-microglial marker, and its expression increases with microglial activation and inflammation, while reactive astrocytes are characterized by the upregulation of GFAP expression. Several studies reported that chronic microglial activation resulted in elevated levels of IL-1β, IL-6, and TNF-α in the striatum and substantia nigra pars compacta of patients with PD [61, 62]. Immunohistochemical analysis of Iba-1 and GFAP showed that the number of activated astrocytes and microglia increased in both the striatum and substantia nigra after MPTP injection, and treatment with our formulation markedly alleviated MPTP-induced glial cell activation. We also found that ASX inhibited the production of proinflammatory mediators, including IL-1β and TNF-α, in the substantia nigra. Regarding the systemic toxicity of the treatments, we observed no unusual changes in the body weight of the mice during the observation period and no significant differences in the body weight at the end of the observations, right before sacrificing them (Supplementary Fig. S11).

Currently, several therapeutic candidates are under clinical trials investigating the role of mitochondrial dysfunction, oxidative stress, and neuroinflammation in the pathogenesis of PD [63]. Given its potent anti-inflammatory and antioxidant properties, our formulation is a promising agent that merits further development toward clinical translation.

In this study, we presented the enhanced neuroprotection against MPTP-induced dopaminergic neurodegeneration of our formulation by facilitating the penetration of ASX by the lactoferrin-conjugated liposomes. Although we have confirmed the accumulation of Lf-LPs by fluorescently labelled LPs, we did not directly evaluate the concentration of ASX in brain. In addition, the further study on other animal models for PD and the careful evaluation of possible toxicity of our formulation might be warranted to verify its neuroprotective efficacy and safety before its clinical translation.

## Conclusion

The Lf-ASX-LP formulation improved the aqueous solubility and stability of ASX, thereby promoting its anti-inflammatory ability both in vitro and in vivo. The functionalization with Lf enhanced the BBB penetration of LPs, improving motor dysfunction and dopaminergic neuron degeneration through anti-neuroinflammatory mechanisms. These encouraging outcomes indicated that our formulation alleviated the clinical symptoms of PD by ameliorating neuroinflammation, oxidative stress, and mitochondrial dysfunction. Our study supports the application of ASX as an anti-PD agent and highlights the potential of liposomal modification and Lf conjugation for improving safety and efficacy.

## Electronic supplementary material

Below is the link to the electronic supplementary material.

Supplementary Material 1

## Data Availability

No datasets were generated or analysed during the current study.
