# Supplementary material for: Astaxanthin-loaded brain-permeable liposomes for Parkinson’s disease treatment via antioxidant and anti-inflammatory responses
Source: J Nanobiotechnology. 2025 Feb 4;23:78. doi: 10.1186/s12951-025-03104-8 (PMC11792324; doi:10.1186/s12951-025-03104-8)
Supplement: Supplementary file 1 — Supplementary Material 1 [file 12951_2025_3104_MOESM1_ESM.docx]

*Supplementary material for*

**Astaxanthin-loaded brain-permeable liposomes for Parkinson’s disease treatment via antioxidant and anti-inflammatory responses**

Thai-Duong Nguyen^1^, Shristi Khanal^2^, Eunhee Lee^1^, Jinsol Choi^1^, Ganesh Bohara^2^, Nikesh Rimal^2^, Dong-Young Choi^2,*^, Soyeun Park^1,*^

*^1^ College of Pharmacy, Keimyung University, 1095 Dalgubeoldae-Ro, Dalseo-Gu, Daegu 42601, Republic of Korea*

*^2^ College of Pharmacy, Yeungnam University, 280 Daehak-Ro, Gyeongsan, Gyeongbuk 38541, Republic of Korea*

**Correspondence:* dychoi@yu.ac.kr (D.-Y.C.); sypark20@kmu.ac.kr (S.P.)

**Supplementary figures**

**
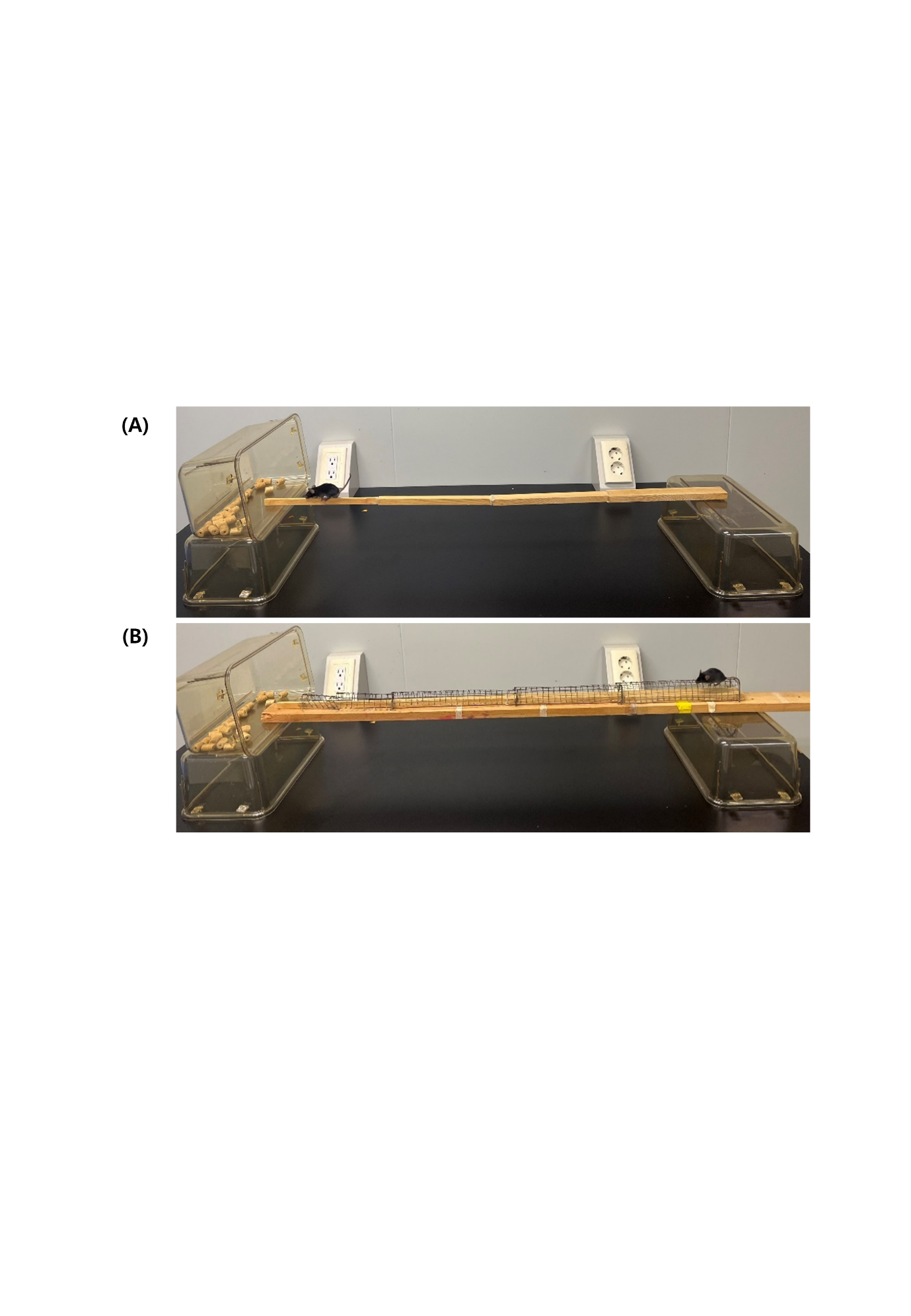
**

**Supplementary Fig. S1.** Equipment set up for (A) beam walking and (B) challenging walking tests.

**
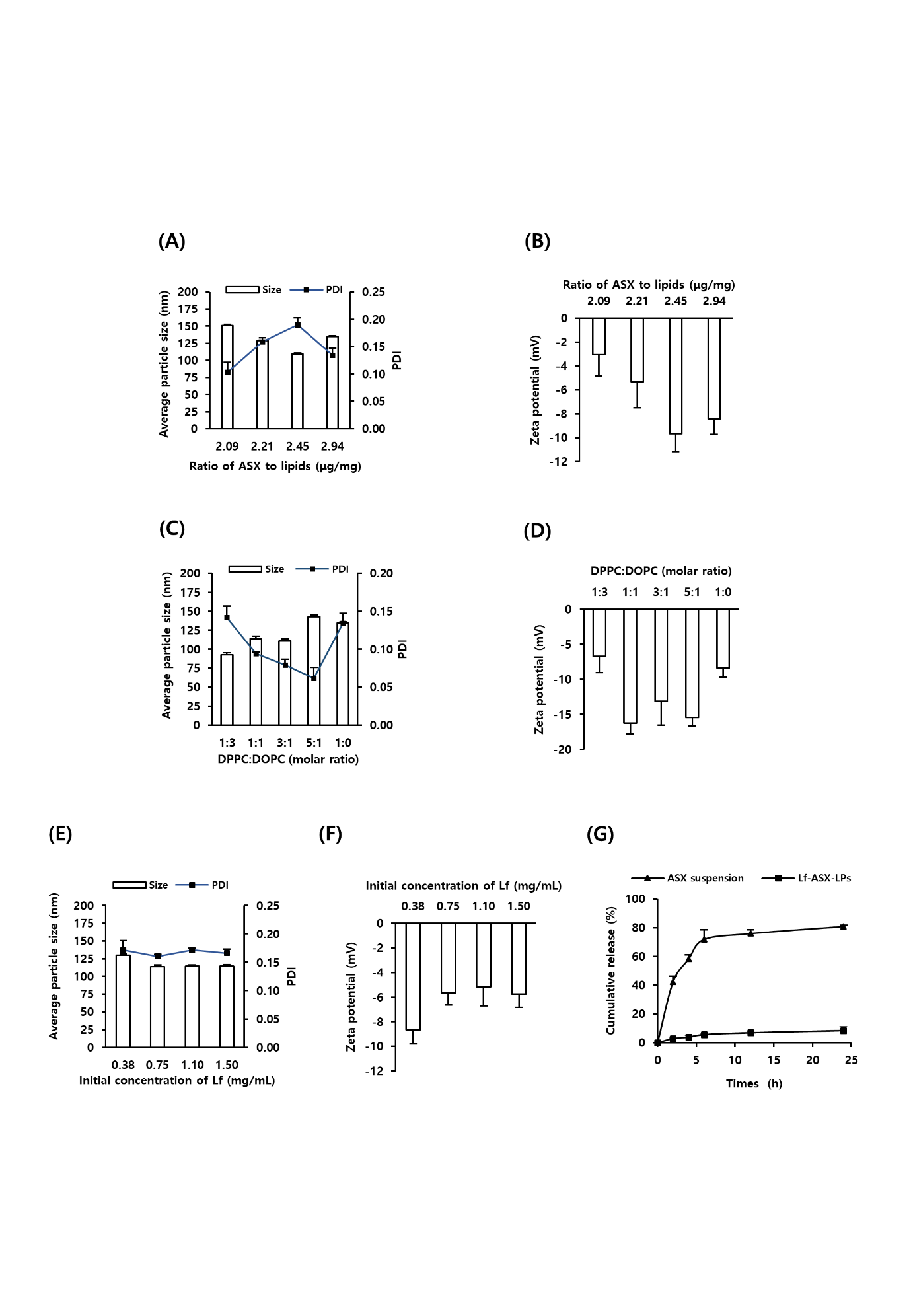
**

**Supplementary Fig. S2.** Characterization of LPs during optimization of formulization. (A, C, E) Average particle size, PDI, and (B, D, F) zeta potential of LPs under varying (A, B) ASX-to-lipid ratios, (C, D) lipid compositions, and (E, F) initial Lf concentrations. (G) Release profile of ASX from Lf-ASX-LPs at pH 7.4.

**
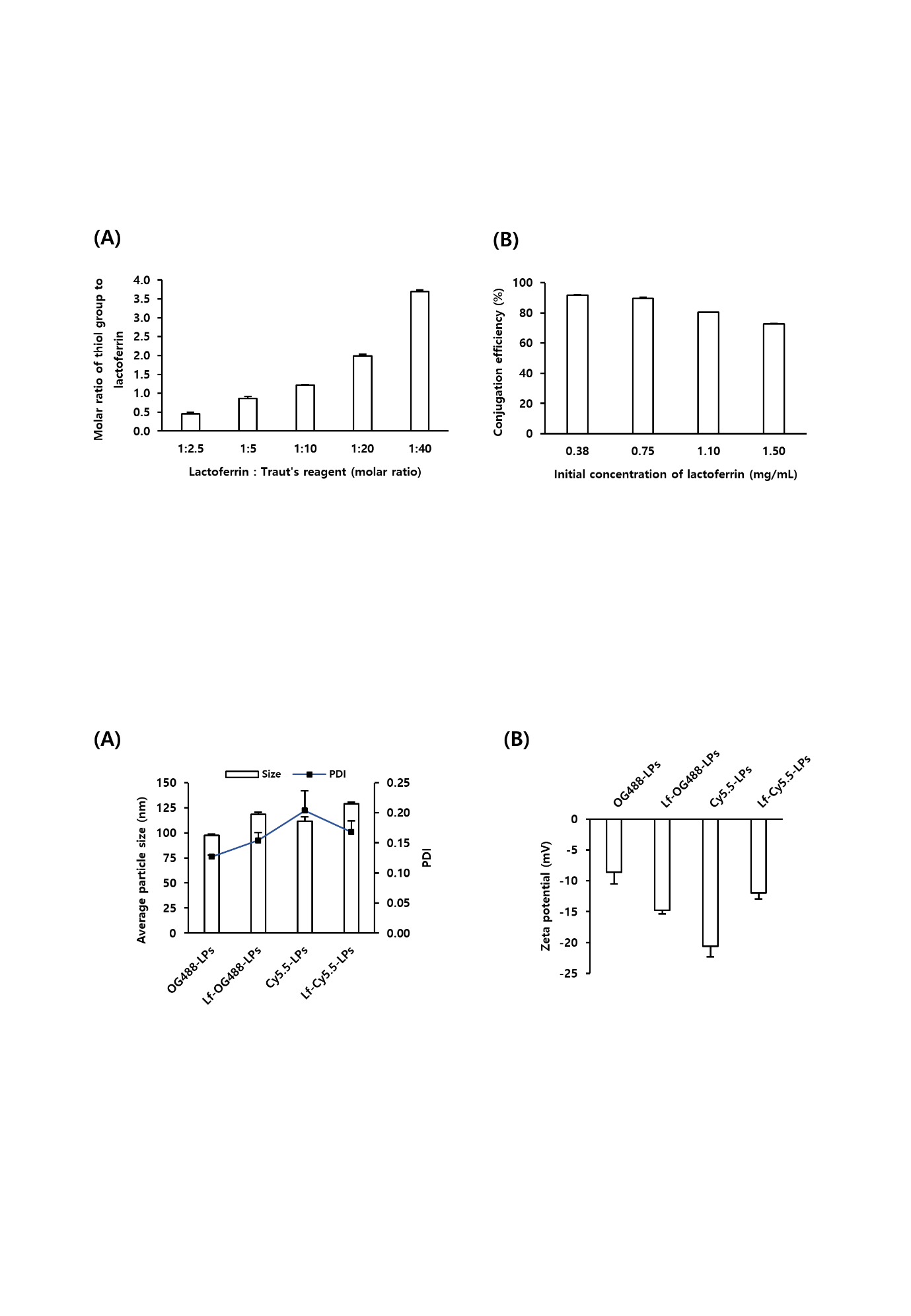
**

**Supplementary Fig. S3.** Optimization of Lf conjugation. (A) Thiolation efficiency of lactoferrin with various molar ratios of Traut’s reagent. (B) Conjugation efficiency under varying initial Lf concentrations.

**
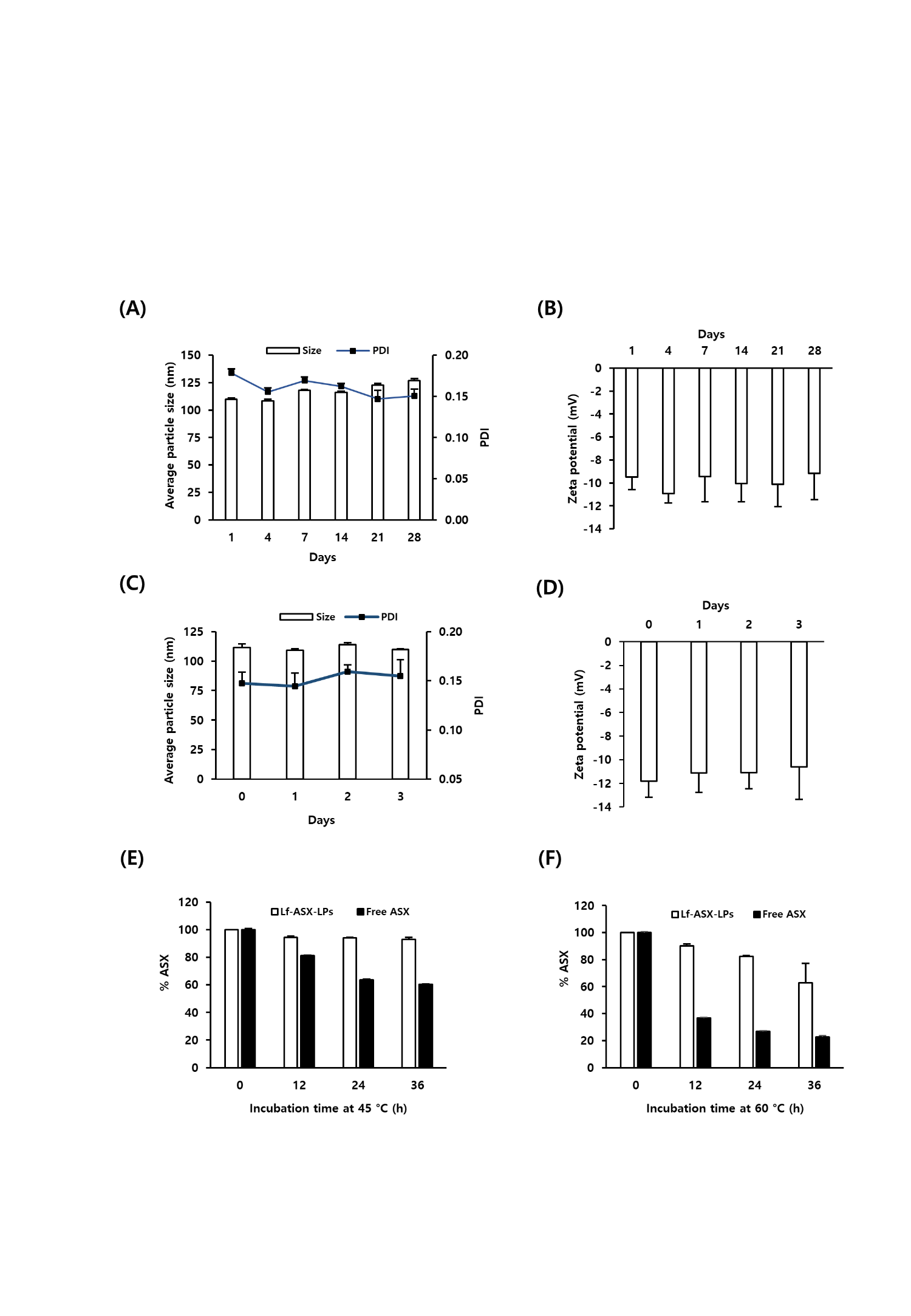
**

**Supplementary Fig. S4.** Physical and chemical stability of Lf-ASX-LPs and ASX. (A, C) Average particle size, PDI, and (B, D) zeta potential of Lf-ASX-LPs stored at 4 °C for 28 days (A-B) and 37 °C for 3 days (C-D), respectively. (E, F) Chemical stability assessed using the ASX concentration from free ASX and Lf-ASX-LPs incubated at (E) 45 °C and (F) 60 °C for 36 h.

**
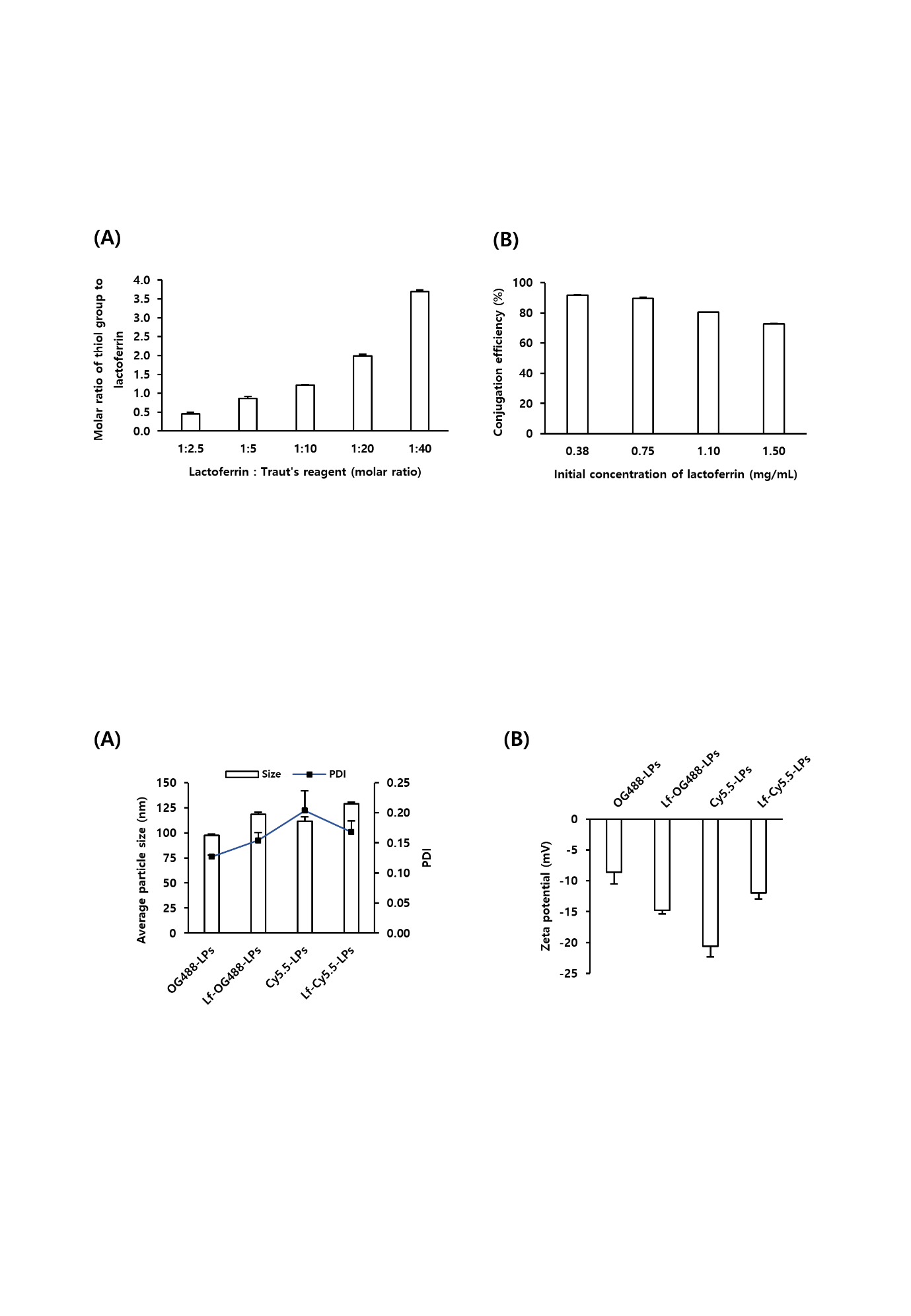
**

**Supplementary Fig. S5.** Physical properties of LPs for Transwell^®^ and biodistribution studies. (A) Average particle size, PDI, and (B) zeta potential of OG488-LPs, Lf-OG488-LPs, Cy5.5-LPs, and Lf-Cy5.5-LPs.

**
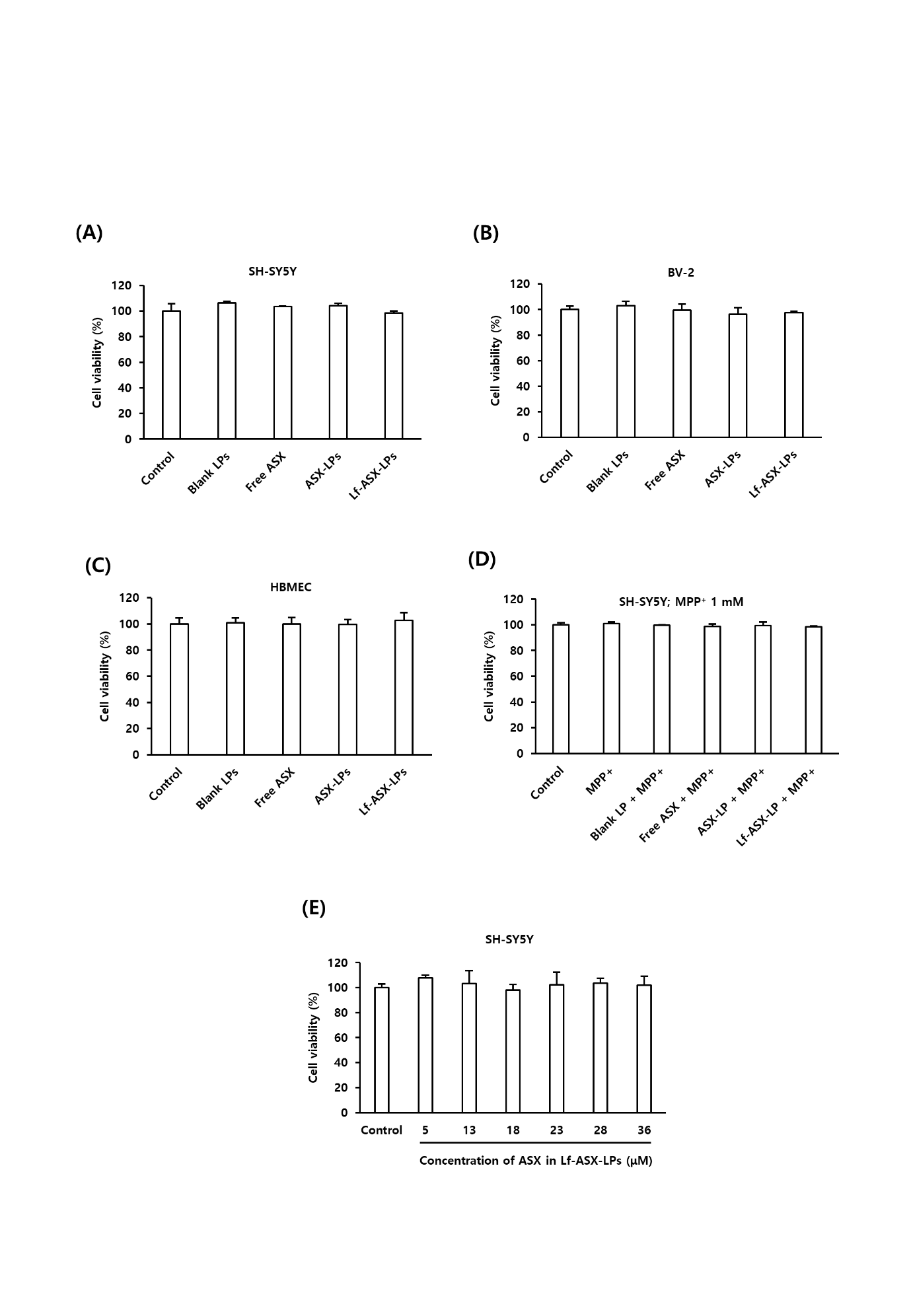
**

**Supplementary Fig. S6.** Cell viability based on MTT assay. Viability of (A) SH-SY5Y cells, (B) BV-2 cells, and (C) HBMECs after treatment with blank LPs, free ASX, ASX-LPs, or Lf-ASX-LPs. (D) Viability of SH-SY5Y cells pretreated with the indicated agents before exposure to 1 mM MPP^+^ for 24 h. (E) Viability of SH-SY5Y cells treated with Lf-ASX-LPs at various ASX concentrations.


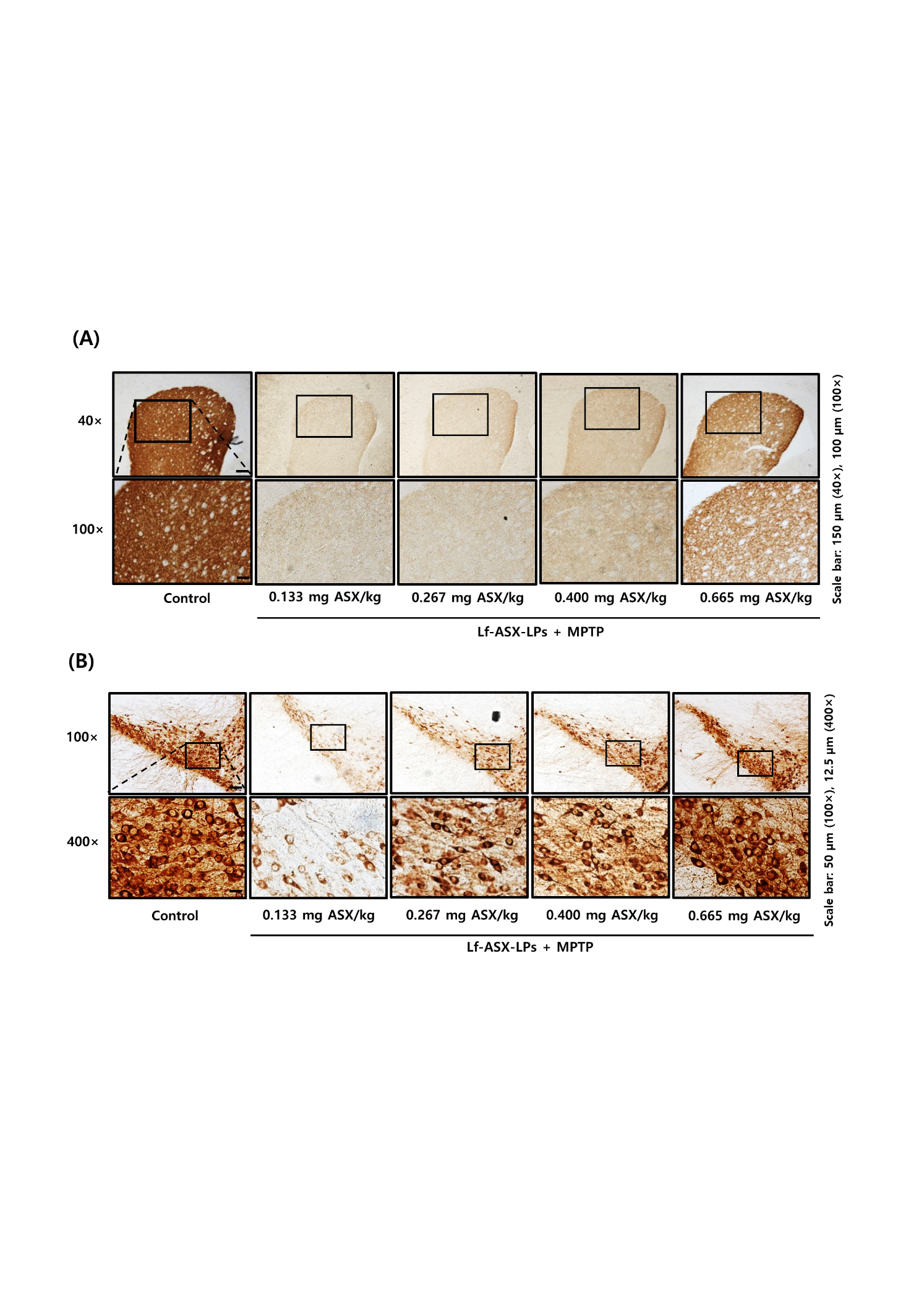


**Supplementary Fig. S7.** Dose-dependent effect of Lf-ASX-LPs in TH neurons of MPTP-treated mice. Immunostaining of (A) TH^+^ fibers in striatum and (B) TH^+^ dopaminergic neurons in substantia nigra.


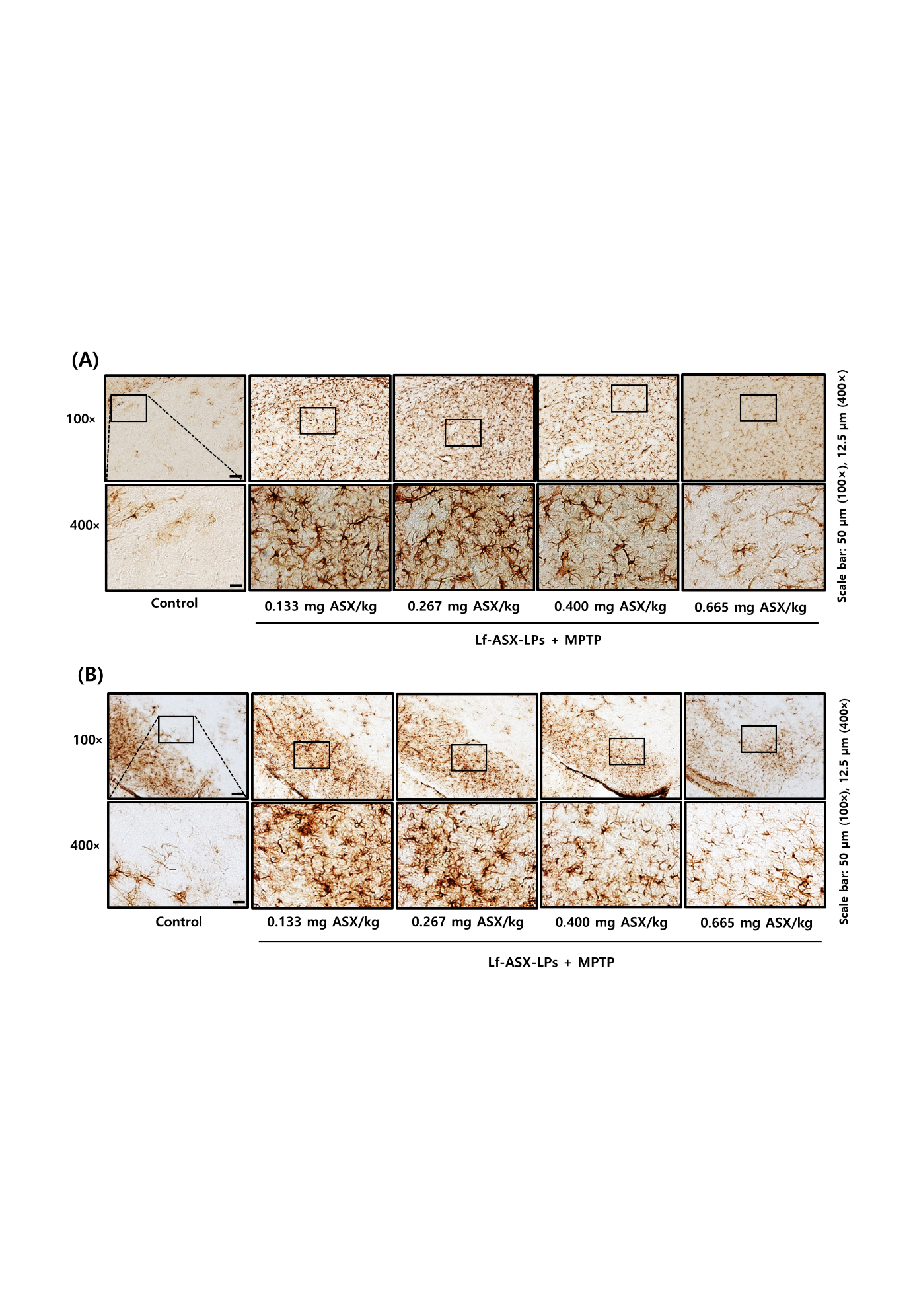


**Supplementary Fig. S8.** Dose-dependent effect of Lf-ASX-LPs on astrocyte activation in MPTP-treated mice. Immunostaining for GFAP in (A) striatum and (B) substantia nigra.

**
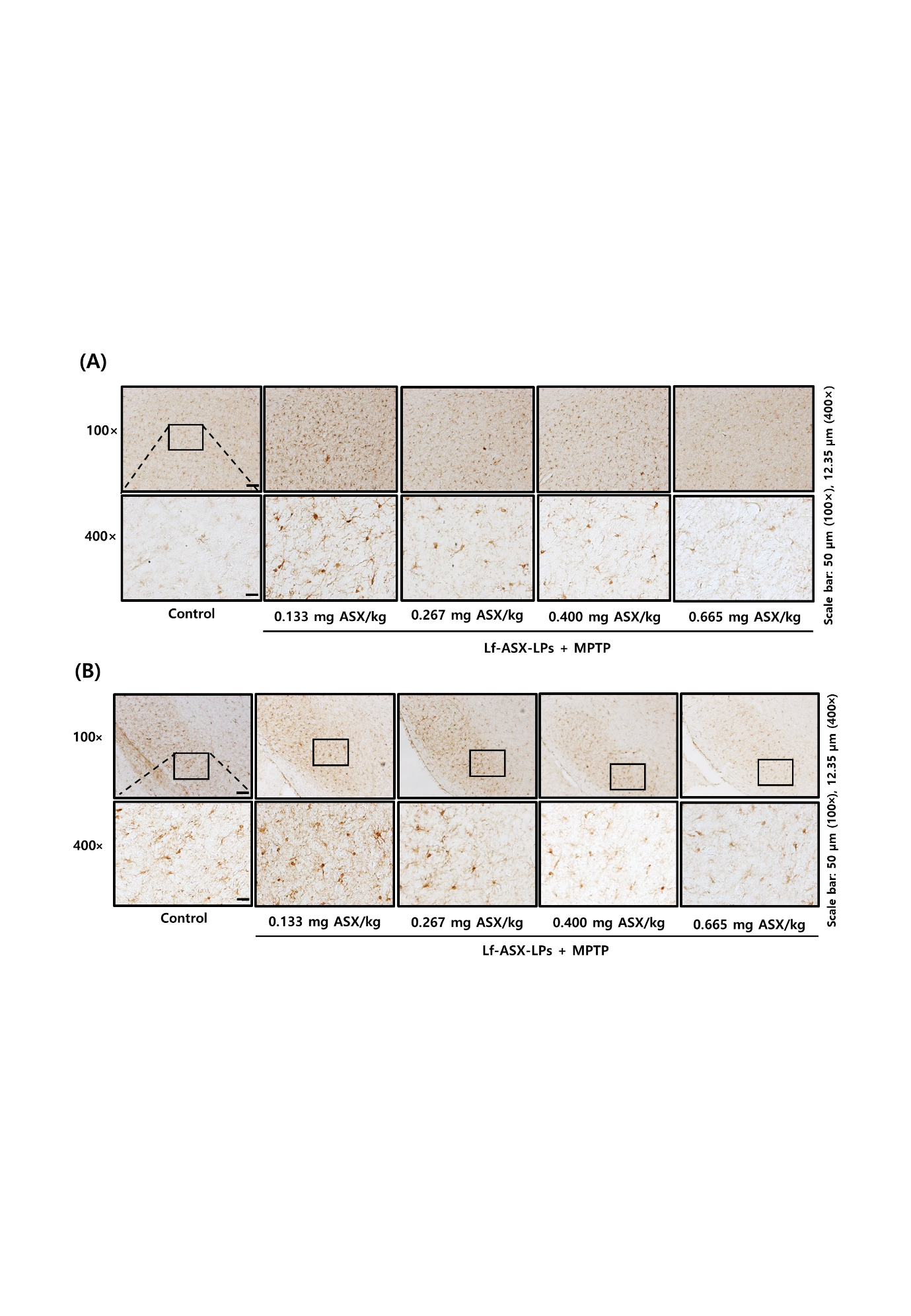
**

**Supplementary Fig. S9.** Dose-dependent effect of Lf-ASX-LPs on microglial activation in MPTP-treated mice. Immunostaining of Iba-1 in (A) striatum and (B) substantia nigra.

**
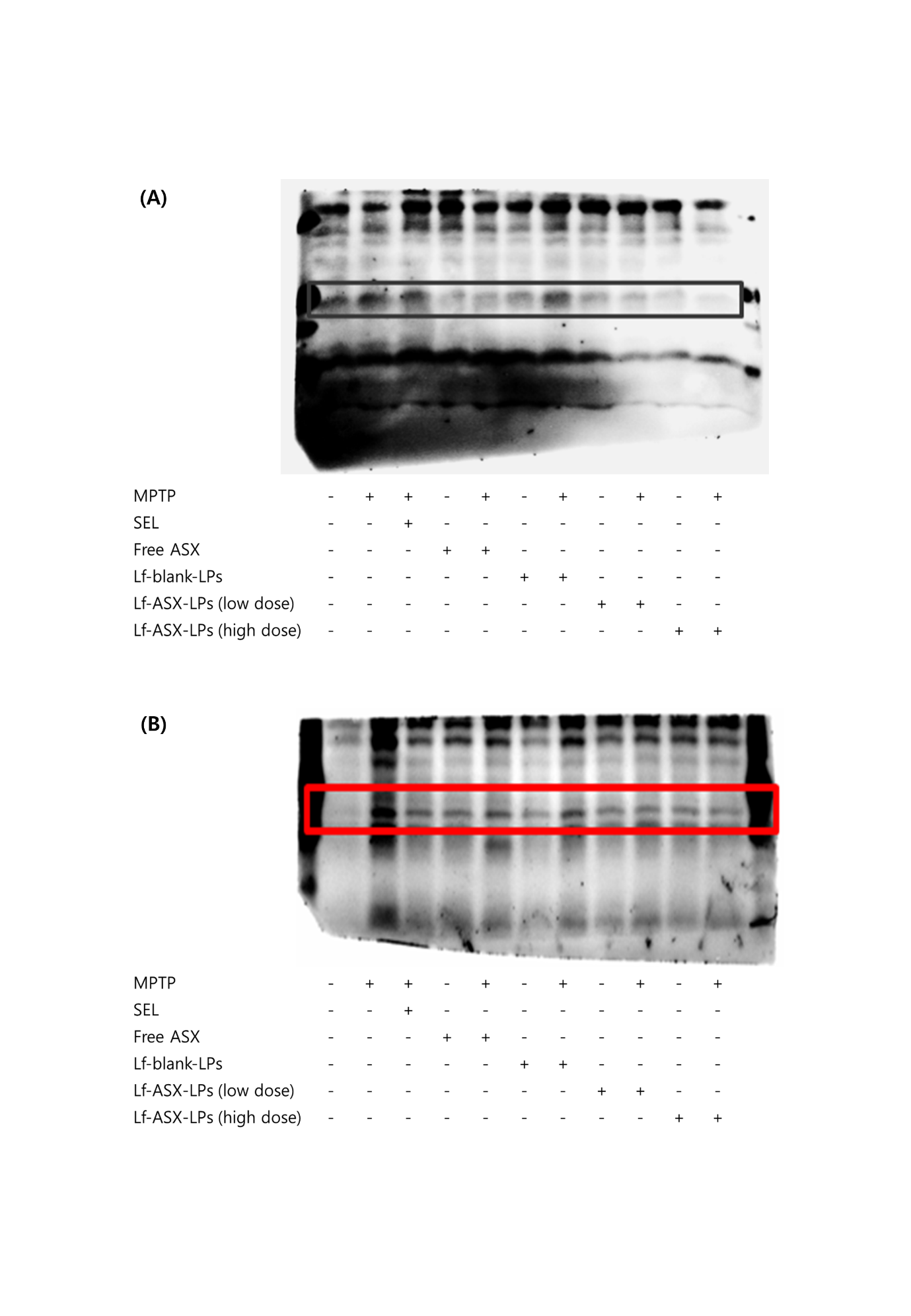
**

**Supplementary Fig. S10.** Western blot analysis of proinflammatory cytokines within substantia nigra. Full blots of (A) IL-1β and (B) TNF-α.


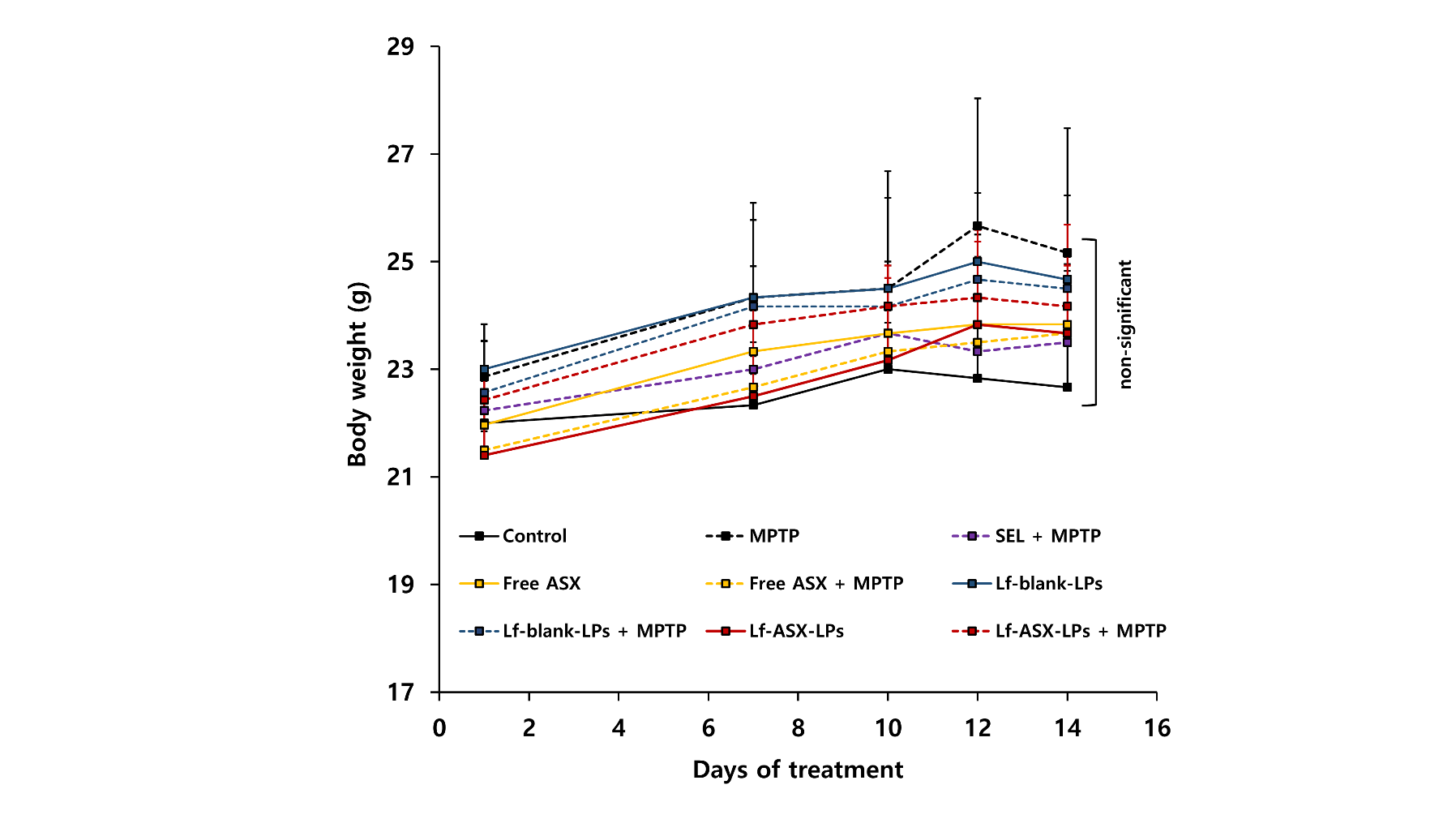


**Supplementary Fig. S11.** Changes in the body weight of mice over the treatment period. The average body weight of each group was calculated, and the error bar represents the standard deviation. There was no statistical difference between the tested groups according to the ANOVA test.
